# Which factors preceding dementia identification impact future healthcare use trajectories: multilevel analyses in administrative data

**DOI:** 10.1186/s12877-023-04643-1

**Published:** 2024-01-23

**Authors:** Anaïs Couret, Maryse Lapeyre-Mestre, Eugénie Gombault-Datzenko, Axel Renoux, Hélène Villars, Virginie Gardette

**Affiliations:** 1 Agence Régionale de Santé Occitanie, Toulouse, France; 2 Maintain Aging Research team, CERPOP, Université de Toulouse, Université Paul Sabatier, Inserm, Toulouse, France; 3 Faculté de médecine, 37 allées Jules Guesde, Toulouse, 31000 France; 4 Department of Pharmacology, Centre Hospitalier Universitaire de Toulouse, Toulouse, France; 5 Centre d‘Investigation Clinique 1436, Team PEPSS “Pharmacologie En Population cohorteS et biobanqueS“, Centre Hospitalier Universitaire de Toulouse, Université Paul Sabatier, Inserm, Toulouse, France; 6 Department of Medical Information (DIM), Centre Hospitalier Universitaire de Toulouse, Toulouse, France; 7 Centre Hospitalier Universitaire de Toulouse, Toulouse, France; 8 Geriatric Department, Centre Hospitalier Universitaire de Toulouse, Toulouse, France; 9 Department of Epidemiology and Public Health, Centre Hospitalier Universitaire de Toulouse, Toulouse, France

**Keywords:** Alzheimer’s disease, Dementia, Healthcare use, Administrative database

## Abstract

**Background:**

Healthcare use patterns preceding a diagnosis of Alzheimer’s Disease and Related Diseases (ADRD) may be associated with the quality of healthcare use trajectories (HUTs) after diagnosis. We aimed to identify determinants of future favorable HUTs, notably healthcare use preceding ADRD identification.

**Methods:**

This nationwide retrospective observational study was conducted on subjects with incident ADRD identified in 2012 in the French health insurance database. We studied the 12-month healthcare use ranging between 18 and 6 months preceding ADRD identification. The five-year HUTs after ADRD identification were qualified by experts as favorable or not. In order to take into account geographical differences in healthcare supply, we performed mixed random effects multilevel multivariable logistic regression model to identify determinants of future favorable HUTs. Analyses were stratified by age group (65–74, 75–84, ≥ 85).

**Results:**

Being a woman, and preventive and specialist care preceding ADRD identification increased the probability of future favorable HUT, whereas institutionalization, comorbidities, medical transportation and no reimbursed drug during [-18;-6] months decreased it. Besides, some specificities appeared according to age groups. Among the 65–74 years subjects, anxiolytic dispensing preceding ADRD identification decreased the probability of future favorable HUT. In the 75–84 years group, unplanned hospitalization and emergency room visit preceding ADRD identification decreased this probability. Among subjects aged 85 and older, short hospitalization preceding ADRD identification increased the probability of future favorable HUTs.

**Conclusion:**

Regular healthcare use with preventive and specialist care preceding ADRD identification increased the probability of future favorable HUTs whereas dependency decreased it.

**Supplementary Information:**

The online version contains supplementary material available at 10.1186/s12877-023-04643-1.

## Background

Over 55 million people suffer from Alzheimer’s Disease and Related Diseases (ADRD) worldwide [1]. ADRD symptoms are based on cognitive decline and functional impairment, with possible fleeting behavioral and psychological symptoms of dementia (BPSD) [2]. A timely diagnosis is recommended to ensure an effective medical management [3]. However, ADRD diagnosis may be complicated by various reasons, including difficulties to access care, stigma and fatalism from both health professionals and patients [4]. Late ADRD diagnosis may lead to unfavorable outcomes [5], such as avoidable emergency care, or non-recommended benzodiazepine use. On the contrary, regular healthcare use is recommended to manage the large variety of ADRD symptoms [6], while ensuring a medical follow-up of other concomitant comorbidities. However, literature investigating factors associated with favorable or unfavorable trajectories studied through several dimensions of healthcare use, and during long follow-up periods is scarce. Although some studies investigated this aspect, they often focused only on a portion of healthcare use (drug dispensing, hospitalization) [7–14]. To our knowledge, none studied the association between healthcare use preceding ADRD identification and future healthcare use trajectories (HUTs). Hence, identifying which healthcare use preceding ADRD identification could be more prone to future favorable HUTs is of interest.

Thus, we aimed to study the determinants of future favorable HUTs, including healthcare use preceding ADRD identification.

## Methods

### Data source

We used data from the FRA-DEM cohort, an open dynamic cohort gathering all subjects identified with incident ADRD since 2011 in the French health insurance database (‘Système National des Données de Santé’, SNDS) (Data Protection French Authority (CNIL) authorization N°1,631,786-DE-2013-037). A wide variety of questions were addressed through the study of data from this national cohort [15–18]. All reimbursed healthcare uses are recorded in the SNDS: ambulatory care, hospital care (with diagnosis), drug dispensing. The SNDS also contains a Long-Term Disease (LTD) registry, which gathers medically confirmed chronic conditions and allows full coverage for related healthcare use. All these information are linked in an anonymous way, and the RESID-EHPAD database providing data regarding Nursing Home (NH) stays [19] was also provided for the FRA-DEM cohort. In the FRA-DEM cohort, subjects with incident ADRD are identified if they met at least one of the three following criteria: (1) two reimbursements of anti-dementia drugs (anticholinesterase inhibitors or memantine) during the year, (2) ADRD hospital diagnosis (ICD-10 codes: “F00-F03”, “G30”, “G31” except “G31.2” and “G31.8”), (3) ADRD LTD registration. As explained above, a LTD registration allows full coverage for healthcare use related to medically confirmed chronic conditions. Thus, some patients with ADRD may be fully reimbursed for their healthcare use related to ADRD (this requires the General Practitioner (GP) or the specialist to request to the French Health Insurance the patient’s ADRD diagnosis in the LTD registry).

### Study population and design

In a preliminary step, which was has been extensively described elsewhere [18], we studied the HUTs of subjects with incident ADRD aged 65 years and more identified in 2012 in the FRA-DEM cohort (Additional file 1). These subjects were clustered using partitioning around medoids applied to Levenshtein distances according to their five-year HUT following ADRD identification in various healthcare services (ambulatory care, hospital care, drug dispensing, institutionalization). A back engineering process by experts (GPs, nurses, neurologists, geriatricians) qualified these clusters as favorable or not, taking into account ADRD guidelines. HUTs referred to the use of the healthcare system during five years after ADRD identification according to guidelines, considering the comorbidity profile of each HUT cluster and comparing the temporal trends in healthcare uses of each HUT cluster with the trends in the average HUT cluster of each age group (Additional file 1).

**Fig. 1:**
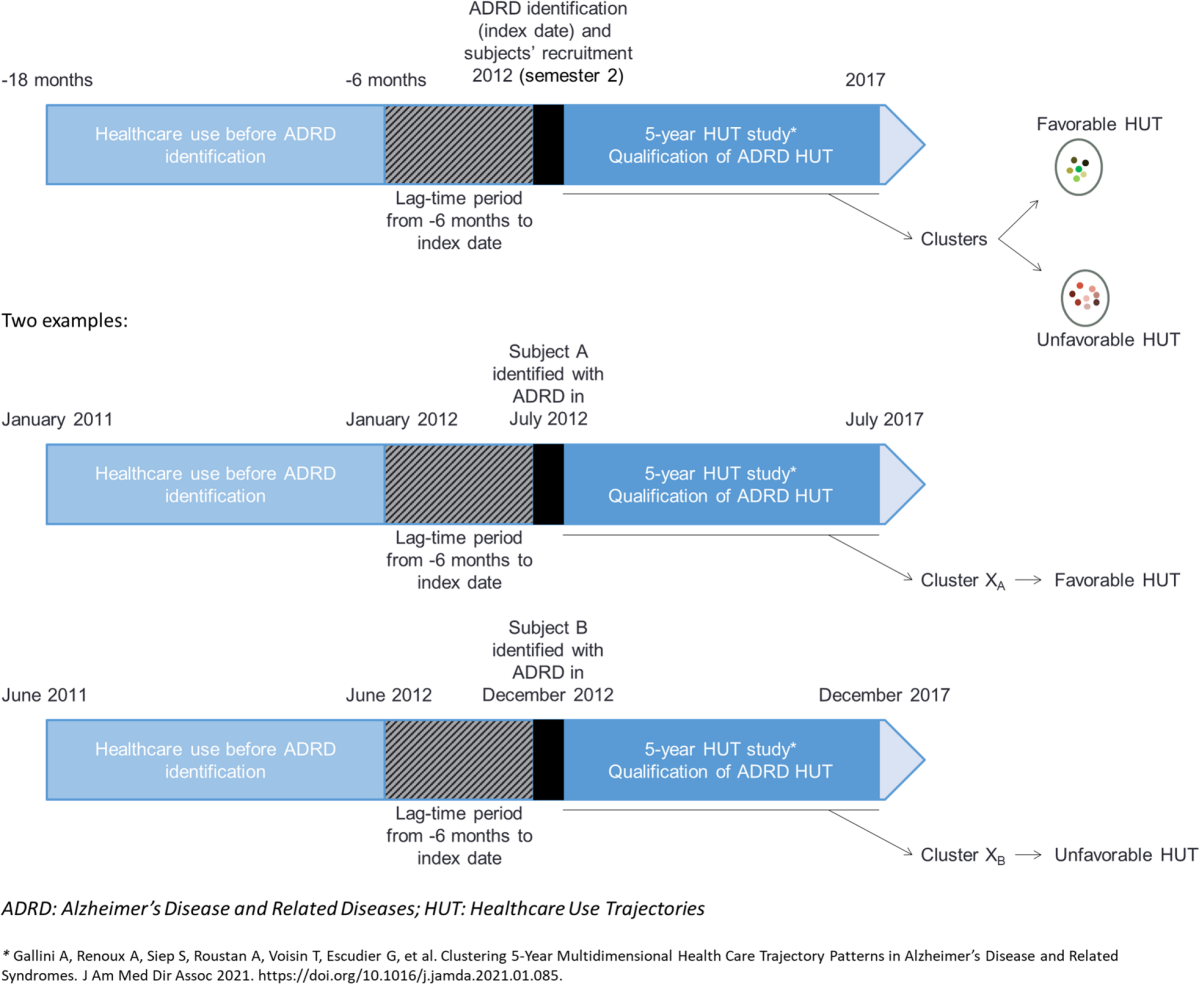
Study design with two examples (subject A and subject B)

Concerning the present study, we chose to focus on a 1-year healthcare use period preceding ADRD identification ([-18;-6] months). We used a 6-month lag-time period preceding ADRD identification, since ADRD diagnosis may precede ADRD identification in administrative data. Data being unavailable before January 2011 and in order to cover an entire year of follow-up, we focused on subjects identified during the second semester of 2012. Fig. 1 described the study design.

### Variables

Socio-demographics variables were studied:

- sex,
- age (continuous),
- a measure of ecological socio-economic level [ 20],
- rural or urban type of the place of stay.

Then, number of comorbidities (continuous) as of December 31, 2012 [21] was studied.

Lastly, we used various variables describing healthcare use preceding ADRD identification during this 1-year period. Details on variables’ construction and categorization are available in Additional file 2. We used consensual definitions for potentially avoidable hospitalization [22, 23], Potentially Inappropriate Medication (PIM) [24], excessive polypharmacy [25]. The variables cover a wide range of healthcare use settings:

- ambulatory visits and medical transportation,
- ambulatory drug exposure,
- medical devices,
- hospitalization,
- institutionalization [ 19].

### Statistical analyses

Factors associated with future favorable HUTs were investigated among 3 age groups (65–74, 75–84, 85 years and older), using a mixed random effects multilevel multivariable logistic regression model. Aging healthcare supply is organized at the department level in France (i.e. number of beds in NH). In continental France, there are 94 departments with a population density ranging from 14.8 inhabitants/km² to 454.1 inhabitants/km². Therefore, a department level was considered as level 1 to take into account geographical differences in healthcare organization. Intra and inter-departmental variances were estimated. The factors included in the multivariate multilevel model (level 2) were of three types: (1) socio-demographics, (2) number of comorbidities and (3) healthcare use preceding ADRD identification. Those factors were included in the multivariate multilevel model in a forward stepwise selection using Akaike Information Criterion. Associations were estimated and presented as adjusted Odds Ratios (aOR) and their 95% Confidence Intervals (95%CI). We verified the linearity assumptions for continuous variables and the residuals’ normality. Analyses were conducted using Stata 17.0 (StataCorp LP, College Station, TX). The significance level was < 0.05.

### Sensitivity analyses

To ensure the validity of our results, we performed two sensitivity analyses. First, we excluded subjects belonging to clusters that the experts were unable to qualify with certainty as favorable or not future HUTs, leading to the exclusion of 845, 4372 and 2617 subjects in the 65–74, 75–84 and 85 and older groups respectively (17.7%, 26.6% and 16.6% respectively). Second, as the institutionalization being expected to have a strong effect on the future HUTs, institutionalized subjects were removed (158, 617 and 1651 subjects excluded in the 65–74, 75–84 and 85 and older groups respectively, representing respectively 3.3%, 3.8% and 10.5% of the subjects).

## Results

**Fig. 2:**
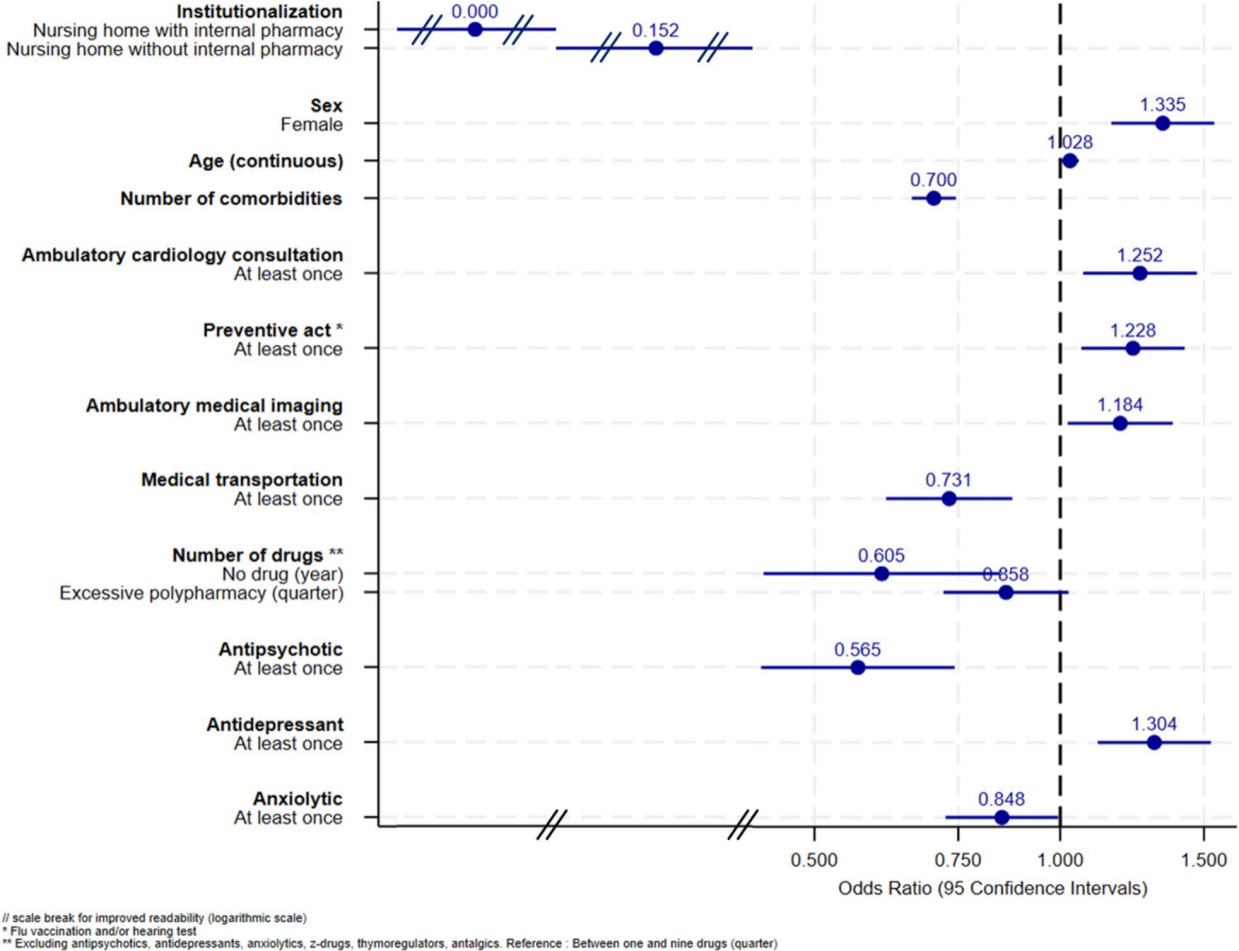
Forestplot of the multilevel multivariate analysis of factors associated with future favorable HUTs (65–74 years group, *n* = 4,764)

**Fig. 3:**
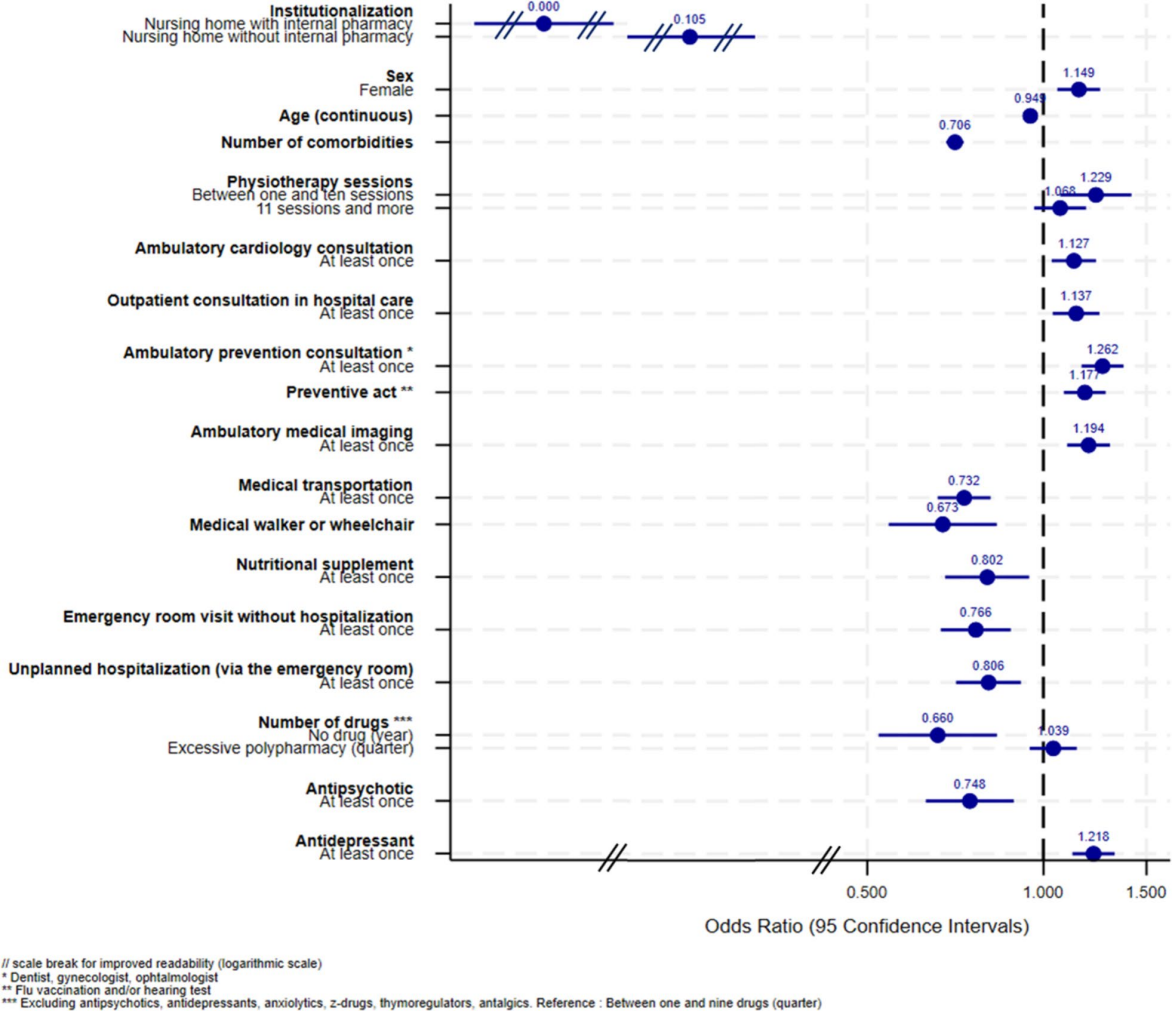
Forestplot of the multilevel multivariate analysis of factors associated with future favorable HUTs (75–84 years group, *n* = 16,441)

**Fig. 4:**
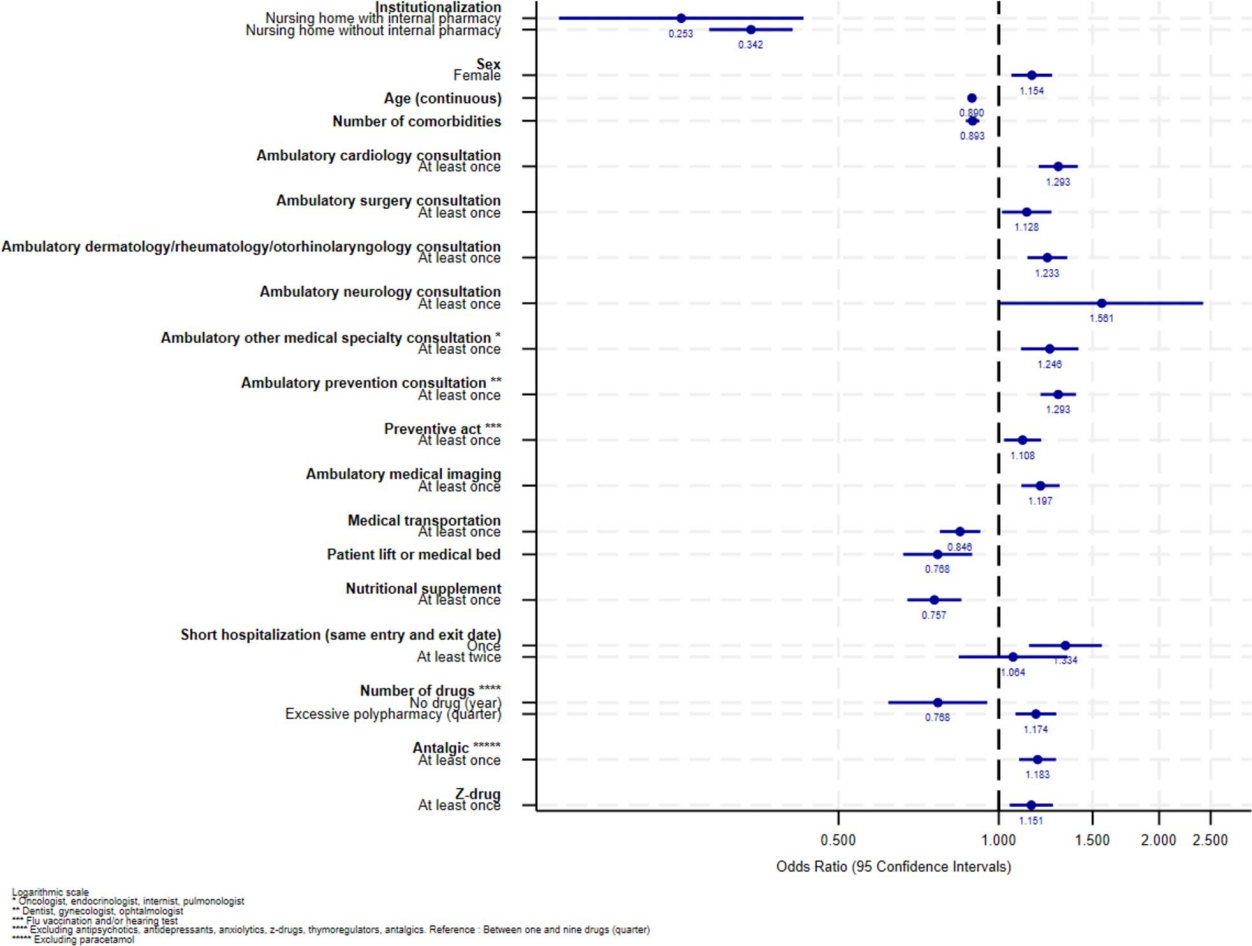
Forestplot of the multilevel multivariate analysis of factors associated with future favorable HUTs (85 and older group, *n* = 15,785)

A total of 36,990 subjects were included in this study. The description of the sociodemographic characteristics and healthcare use preceding ADRD identification of the study population by age group and according to their favorable (or not) future HUT is available in Additional file 3. The part of inter-departmental variance in total variance was small but statistically significant in all age groups (2.0%, 2.9% and 0.9% for the 65–74, 75–84 and 85 and older groups respectively). The results of the multivariate analyses are presented in Figs. 2, 3 and 4 for the 65–74, 75–84 and 85 and older groups respectively and in Additional files 4, 5 and 6.

There were similarities between the three age groups. **Institutionalization** (in NH with or without internal pharmacy) preceding ADRD identification was strongly negatively associated with future favorable HUTs for all age groups (for example, 95% Confidence Intervals (CI) 65–74 years group: NH with internal pharmacy [1.22E-08–3.21E-06], NH without internal pharmacy [0.06–0.42]). In the 65–74 years group, youngest subjects had a decreased probability of future favorable HUTs (95%CI [1.00-1.05] for increasing age), while older age decreased this probability in the other two age groups (95%CI [0.94–0.96] and [0.88–0.90] for the 75–84 and 85 and older groups respectively). In all age groups, **a high number of comorbidities** (95%CI [0.66–0.75], [0.68–0.73] and [0.87–0.92] for the 65–74, 75–84 and 85 and older groups respectively), **the absence of reimbursed drug dispensing** (95%CI [0.43–0.84], [0.52–0.83] and [0.61–0.93] for the 65–74, 75–84 and 85 and older groups respectively) and **the use of medical transportation** (for example, 95%CI 65–74 years group: [0.61–0.87]) decreased the probability of future favorable HUTs. On the contrary, in all age groups, being **female** (95%CI [1.16–1.54], [1.07–1.25] and [1.07–1.27] for the 65–74, 75–84 and the 85 and older groups respectively), **ambulatory cardiology consultation** (95%CI [1.07–1.47], [1.03–1.23] and [1.18–1.39] for the 65–74, 75–84 and 85 and older groups respectively), **medical imaging** (for example, 95%CI 65–74 years [1.02–1.37]) and **preventive acts** (for example, 95%CI 65–74 years [1.06–1.42]) increased the probability of future favorable HUTs. Some determinants were identified in 2 age groups. In the 65–74 and 75–84 years groups, **antidepressant** dispensing (95%CI [1.11–1.53], [1.12–1.32] respectively) preceding ADRD identification increased the probability of future favorable HUTs, while **antipsychotic** dispensing (95%CI [0.43–0.74], [0.63–0.89] respectively) decreased this probability. In those age groups, **excessive polypharmacy** during a quarter preceding ADRD identification was not significantly associated with future favorable HUTs, while it increased this probability in the 85 and older group (95%CI [1.09–1.30]). In the 75–84 and 85 and older groups, **prevention consultation** (95%CI [1.16–1.37], [1.19–1.39] respectively) preceding ADRD identification increased the probability of future favorable HUTs, while reimbursement for **nutritional supplement** (95%CI [0.68–0.95], [0.68–0.86] respectively) decreased this probability. Some determinants were observed in a single age group. In the youngest, using **anxiolytic** (95%CI [0.72–0.99]) preceding ADRD identification was significantly associated with a decreasing probability of future favorable HUTs. In the 75–84 years group, between one and ten **physiotherapy sessions** (95%CI [1.07–1.41]) and **outpatient consultation in hospital care** (95%CI [1.04–1.25]) increased the probability of future favorable HUTs, while using the **emergency room without hospitalization** (95%CI [0.67–0.88]), **unplanned hospitalization** (95%CI [0.71–0.91]) and **reimbursement for a medical walker or a wheelchair** (95%CI [0.54–0.83]) decreased this probability. In the oldest age group, **surgery, dermatology/rheumatology/otorhinolaryngology, neurology, other medical specialty consultations** (95%CI [1.01–1.26], [1.01–2.44], [1.13–1.34], [1.11–1.42] respectively), **short planned hospitalization** (95%CI [1.14–1.57]), **antalgic and z-drug** dispensing (95%CI [1.10–1.29], [1.05–1.27] respectively) preceding ADRD identification increased the probability of future favorable HUTs. On the contrary, **reimbursement for a patient lift or a medical bed** (95%CI [0.67–0.90]) preceding ADRD identification decreased the probability of future favorable HUTs in the 85 and older group. Sensitivity analyses leading to the exclusion of [1] uncertain clusters (for which the experts were unable to classify with certainty as favorable or not) and [2] institutionalized subjects yielded stable results (data not shown).

## Discussion

This research provides unique information regarding healthcare use preceding ADRD identification in the French healthcare reimbursement database and its association with future favorable HUTs. This study allows a global understanding of the individual characteristics of the subjects, as well as healthcare use (quantitative and qualitative) preceding ADRD identification, while taking into account geographical disparities in healthcare supply.

Individual factors (such as institutionalization or number of comorbidities) had the strongest effect on the probability of future favorable HUTs, followed by healthcare use preceding ADRD identification, and geographic variations had the lowest effect. These geographic variations could be explained by geographic disparities in the accessibility to healthcare services.

Several factors increasing the probability of future favorable HUTs were identified. In all age groups, being a woman increased it [26, 27]. Women may have better adherence to recommended medical follow-up throughout their lives or better lifestyles with individual health-promoting practices [28], which could be explained by a better health literacy [29–31]. In all age groups, using ambulatory preventive and medical imaging, cardiology consultation increased the probability of future favorable HUTs. Antidepressant dispensing (in the 2 youngest age groups), outpatient consultation and physiotherapy sessions (75–84 years group), using various ambulatory specialists consultations, short planned hospitalization, excessive polypharmacy (85 and older) increased the probability of future favorable HUTs. All these healthcare use factors suggested a regular medical follow-up, which may be enhanced by a GP care coordination for individuals who may be regularly cared by several healthcare providers, and prone to healthy lifestyle, visiting regularly various specialists for preventive care. This pattern suggests an early ADRD detection with further healthcare services’ support shortly after ADRD identification. The presence of informal caregivers enhancing the medical follow-up could also explain this result. Antidepressant dispensing could indicate a medical follow-up for depressive disorders, which may be a prodrome of ADRD or increase the risk of ADRD [32–34]. Therefore, health professionals may pay particular attention to the onset of ADRD symptoms in subjects with depressive disorders, allowing a timely ADRD diagnosis and a possible future healthcare planning.

On the other hand, several factors decreased the probability of future favorable HUTs. In the 65–74 years group, the youngest subjects had a decreased probability of future favorable HUTs, which could be explained by a late diagnosis related to a medical nomadism and difficulties in identifying ADRD in young people [35]. Among subjects aged 75 and older, increasing age may be associated with later suboptimal ADRD management due to health professionals fatalism (alongside family caregivers and subjects themselves) [36], leading to a disengagement towards medical follow-up. An increased number of comorbidities decreased the probability of future favorable HUTs [36]. The presence of concurrent diseases [15, 37] may prevent subjects to be timely diagnosed or access healthcare related to ADRD. Some life-threatening concomitant diseases may put ADRD as of secondary importance, such as cardiac insufficiency. Other comorbidities, such as chronic psychotic disorders (schizophrenia for example), may involve symptoms which may represent barriers to ADRD management or identification [38, 39]. In the 65–74 and 75–84 years groups, antipsychotic dispensing preceding ADRD identification decreased the probability of future favorable HUTs, as well the addition of anxiolytic dispensing in the 65–74 years group. These drug exposures may be related to the preexistence of psychiatric disorders notably psychotic history, as well as undiagnosed BPSD and our data cannot disentangle these two phenomena. Moreover, anxiolytic dispensing in the 65–74 years group preceding ADRD identification could suggest medical nomadism facing prodromal BPSD. This exposure could deteriorate the cognitive functions [40] and degrade the future HUTs. Behavioral disturbances may suggest undiagnosed prodromal BPSD, leading to a late ADRD diagnosis. Factors related to dependency preceding ADRD identification, including institutionalization, need of medical transportation or of medical devices for mobility aids (75–84, 85 and older groups), decreased the probability of future favorable HUTs in all age groups. This dependency-related healthcare use could suggest a reduced network of informal caregivers, making at-home stay or access to ambulatory consultation more complex [41]. Institutionalization does not rectify a reduced network of informal caregivers, the medical follow-up of residents remaining under the responsibility of the regular GP. Moreover, in order to reduce the burden of dependency on healthcare use, secondary or even tertiary prevention of dependency could be implemented among institutionalized subjects with ADRD. The presence of comorbidities could enhance the burden of dependency. All these aspects of dependency could complicate access to healthcare use, with the physical, environmental or material barriers that they constitute [41]. Moreover, the presence of dependency could constitute a symptom of undiagnosed ADRD and therefore, indicate a late ADRD diagnosis.

In the 75–84 years group, subjects having emergency room visits followed by an hospitalization or not preceding ADRD identification were less likely to have future favorable HUTs [42]. Those unplanned healthcare uses could reflect a lack of medical follow-up and an unidentified ADRD onset. It could also suggest that subjects were less adherent to the recommended medical follow-up or were followed by a GP with suboptimal practices. It has been shown in literature that emergency room visits increased the risk of autonomy loss and death [43], which could lead to erratic HUTs. Moreover, the absence of drug dispensing during the year (no reimbursed drug) preceding ADRD identification decreased the probability of future favorable HUTs, which supports the previous interpretation of a sub-optimal medical follow-up. More than 75% of these subjects did not visit any GP or just once preceding ADRD identification (versus less than 5% subjects with any drug reimbursement, data not shown).

Literature has regularly shown the role of socioeconomic deprivation as a determinant for poor access to health care and health outcomes [20, 44–47], but the deprivation index did not appear as a significant determinant for future favorable HUTs in our analysis. The existence of universal health insurance in France could explain this finding, as well as institutionalization less subject to financial constraints in France compared to other countries. Deprivation index is a proxy for individual socioeconomic deprivation and could not perfectly reflect the individual socioeconomic status. The number of GP consultations within the year preceding ADRD identification was not associated with future favorable HUTs, probably because it was counterbalanced by the high burden of comorbidities on future HUTs. The duration of GP consultations could be a better measurement of healthcare coordination by a GP but this variable was unavailable in our database.

To our knowledge, this study is the first to investigate factors of healthcare use preceding ADRD identification associated with future favorable HUTs, taking into account geographical differences. Although there is data studying healthcare use of subjects preceding their ADRD identification, it is compared to healthcare use of control subjects [7, 10, 11, 14, 42]. Other studies investigated longitudinal trend of healthcare use preceding ADRD identification [9, 12, 13, 48] but did not confront it with healthcare use after diagnosis.

Our study presents limitations. First, the SNDS does not contain information regarding the presence of family caregivers, BPSD, ADRD etiology or severity, living arrangement or lifestyle habitus, which may impact healthcare use. Hence, those potential confounding factors have not been included in our models. Moreover, other potential confounding factors have not been controlled in our analyses because they can vary according to the healthcare investigated and we privileged a unique model (stratified on the age groups) considering several healthcare uses. Moreover, ADRD definition relied on administrative data in which index date does not always coincide with diagnosis date. This latter could precede the index date, which is why we applied a 6-month time lag period [7]. Second, the studied follow-up preceding ADRD identification was restricted to 1 year because of data availability. Then, the use of consultation with a geriatrician, a health professional involved in the healthcare coordination, cannot be measured in the SNDS. Lastly, drug dispensing was not measurable for subjects living in NH with an internal pharmacy [19] nor during hospitalizations. Having information about drug dispensing, especially psychotropic drug dispensing, during institutionalization or hospitalization could have enabled us to improve the quality of our results. However, the variable “institutionalization” allowed us to take this into account in multivariate analyses and sensitivity analyses retrieved stable results.

Nevertheless, this research has several strengths. First, we used an administrative database, reflecting real life consumption of healthcare, with a good exhaustiveness, regarding consultations and hospitalizations. Moreover, using administrative data allowed us to have little selection and low attrition. Second, our study population were beneficiaries of the ‘Regime General’ of the French insurance health system, which covers around 70% of the French population (36,990 subjects allowing multilevel analyses). We restricted our study population to the ‘Regime General’ beneficiaries to ensure a better quality of data, the quality of data from other health insurance schemes being lower. Third, this study was the first to confront healthcare use preceding ADRD identification and the future 5-year HUTs, with such an extensive approach of healthcare use (ambulatory and hospital care, institutionalization, drug dispensing and medical devices reimbursement). Finally, our sample size allowed us to take into account the geographical and healthcare supply differences using multilevel analysis.

## Conclusions

This study highlighted the importance of an effective regular healthcare use in order to increase the probability of future favorable HUTs. This relies on the prescription of various healthcare by a GP, the availability of such services and its implementation by the patient or his relatives. On the contrary, healthcare use preceding ADRD identification reflecting a possible late ADRD diagnosis decreased this probability. Hence, it would be interesting to conduct qualitative studies in order to identify the reasons for suboptimal coordinating care. Moreover, patient health literacy should be improved, as well as health promotion, so that they can pretend to use various and recommended healthcare. Particular attention should be paid to some profiles at greater risk of delayed diagnosis (younger subjects and subjects with psychiatric comorbidities). The implementation of prevention consultations in recent years could allow a timely diagnosis, which appear necessary to enable future favorable HUT. This study showed a negative association of institutionalization before ADRD identification with the probability of future favorable HUT. This result may suggest the need to invest in secondary or tertiary prevention of loss of autonomy in NH.

### Supplementary Information

**Additional file 1. **Methods of the 5-year Healthcare Use Trajectories study.

**Additional file 2.** Details on the construction and categorization of the healthcare use variables ([-18;-6] months).

**Additional file 3.** Description of sociodemographics characteristics and healthcare use preceding ADRD identification ([-18;-6] months) by age group and according to the future favorable (or not) healthcare use trajectories (HUTs).

**Additional file 4.** Results of the multilevel multivariate analysis of factors associated with future favorable healthcare use trajectories of the 65-74 years group (*n*=4,764).

**Additional file 5.** Results of the multilevel multivariate analysis of factors associated with future favorable healthcare use trajectories of the 75-84 years group (*n*=16,441).

**Additional file 6.** Results of the multilevel multivariate analysis of factors associated with future favorable healthcare use trajectories of the 85 and older group (*n*=15,785).

## Data Availability

The data that support the findings of this study are available from the CNAM (*Caisse Nationale d’Assurance Maladie*) but restrictions apply to the availability of these data, which were used under license for the current study, and so are not publicly available. We are not allowed to share these data due to legal restrictions, but SNDS data are accessible to researchers who meet the criteria for access (request for access is evaluated by Commission Nationale de l’Informatique et des Libertés, https://www.health-data-hub.fr/page/faq-english).
