# Supplementary material for: Which factors preceding dementia identification impact future healthcare use trajectories: multilevel analyses in administrative data
Source: BMC Geriatr. 2024 Jan 23;24:89. doi: 10.1186/s12877-023-04643-1 (PMC10807194; doi:10.1186/s12877-023-04643-1)
Supplement: Supplementary file 1 — Additional file 1. Methods of the 5-year Healthcare Use Trajectories study. [file 12877_2023_4643_MOESM1_ESM.docx]

# Additional file 1: Methods of the 5-year Healthcare Use Trajectories study

This study focused on Healthcare Use Trajectories (HUT) of subjects identified with Alzheimer’s Disease and Related Diseases (ADRD) in 2012 in the FRADEM cohort. The methodology and results have been published elsewhere [1]. First, in each age group (65-74, 75-84, 85 and older), subjects were clustered using partitioning around medoids applied to Levenshtein distances according to their 5-year healthcare use following ADRD identification in various healthcare services. Eleven dimensions of healthcare use were considered to build these HUT:

- Dimension 1: ambulatory medical care
- Dimension 2: ambulatory nursing care
- Dimension 3: ambulatory allied health professional care
- Dimension 4: ADRD drugs
- Dimension 5: antipsychotic drugs
- Dimension 6: hospitalization for ADRD follow-up
- Dimension 7: hospitalization for ADRD or BPSD (Behavioral or Psychiatric Symptoms of Dementia)
- Dimension 8: medical specialists
- Dimension 9: hospitalizations (other than dimensions 6, 7 and 10)
- Dimension 10: emergency
- Dimension 11: institutionalization

Monthly patients’ use was measured for each dimension during 5 years following ADRD identification. In each age group, 15 HUT clusters were retrieved and then, qualified as favorable or not by a group of experts in the field (general practitioners, nurses, neurologists, geriatricians). The qualification of each HUT cluster was realized in three steps:

1. Description of the HUT cluster according to each dimension
2. Ranking of the HUT clusters, from worst to best and according to national and international recommendations
3. Assessment of the favorability of the HUT cluster (yes or no)

During step (1), experts were asked to qualify, dimension by dimension, baseline rates of healthcare use, longitudinal use (enhancement and/or continuity of care), and the characteristics suggested by the cluster (e.g. BPSD, late diagnosis…). The feedback we wanted to receive from the experts was the following:

- Interpretation of healthcare utilization in each dimension, with particular attention given to 2 criteria: (1) initial rates of use (as compared to the whole age class in the same period) and (2) trends in use over time : dimension by dimension, the experts were collectively asked to describe if there was a low/ mean/ high level of use of such services at baseline, and then how use evolved over time (for example : ‘baseline high use of neuroleptics with sustained or decreased use over time…’, ‘low use of dimension x which did not increase over time’, ‘rapid use of nursing homes’).
- Whether the cluster suggested a particular etiology (e.g. frontotemporal, BPSD, late diagnosis)?

If there were no a priori criteria set by the experts suggesting favorable, neutral or unfavorable trajectories in each dimension set by the experts before the sessions, rules emerged consensually during data interpretation, considering the patients’ perspective (see table provided below).

During step (2) of clusters’ ranking, based on a multidimensional assessment of descriptions made in step 1 and the criteria presented, experts ordered clusters from worst to best, on a scale of 0 to 10. Assessments were also based, regardless of survival, on i) a histogram of the mean half-yearly use of each category and ii) cluster summary statistics including the distribution of gender, age, comorbidities, deprivation index and first ADRS identification criterion. There was no predefined cut-off (such as 5/10) considered as defining an unfavorable HUT, experts secondarily defined which scores were considered to reflect favorable HUT. The panel collectively debated on the cluster’s description, scoring and final classification (favorable or not). Disagreements were resolved during the session, through discussion, including several rounds of discussion when necessary, although this was generally not the case. Thereafter, a few days later, conclusions for the 45 clusters were summarized by the researchers and checked for any inconsistencies across age groups and experts were asked for ultimate validation.

Thus, the favorability referred to a multidimensional assessment, relying on the interpretation of 11-dimension healthcare utilization with particular attention given to 2 criteria: (1) initial rates (as compared to the whole age class in the same semester) and (2) their trend over time. The experts’ interpretation also took into account the mean age, sex ratio, comorbidity profile, criteria for ADRD identification (to gauge the reliability of ADRD overdiagnosis), and setting of clusters.

Time to death was also taken into account to qualify the HUT in certain cases. In the youngest age group (65-74 years), and at a lesser extent, in the 75-84 years group, higher death rates occurring in the first semesters in cluster without higher rates of concomitant life-threatening comorbidity could suggest potential late diagnosis and a previous erratic trajectory, particularly in the youngest age group. In that cases, death occurring in the first semesters suggested an unfavorable HUT for experts.

|  | **In favor of a favorable HUT** | **Neutral** | **In favor of an unfavorable HUT** |
| --- | --- | --- | --- |
| **Dimension 1** Ambulatory medical care | Sustained high rates of use and/or increasing use over time, in particular ADRD specialists or long and complex visit | Sustained low use over time | Decreasing use over time |
| **Dimension 2** Ambulatory nursing care | Sustained high rates of use and/or increasing use over time, especially in assistance in activities of daily living or both care | Sustained low use over time | Decreasing use over time ^§A^ |
| **Dimension 3** Other ambulatory allied health professional care (physical therapy and speech therapy) | Sustained high rates of use and/or increasing use over time, especially for speech therapy which may be more specific than physiotherapy | Sustained low use over time  Slight increased use to physiotherapy over time (which may reflect many other indications than ADRD) | Decreasing use over time ^§A^  Decreasing use of speech therapy over time |
| **Dimension 4**  ADRD drugs (Cholinesterase inhibitors, memantine)  ^£^ | Particularly among younger subjects (because of fewer comorbidities and contra indications than the over 85 yo) : increasing use (whether continued or not) of cholinesterase inhibitors or memantine, or both in the first semesters | Particularly among the oldest age classes: flat trajectory | Particularly in younger subjects: no increasing use of cholinesterase inhibitors or memantine, or both in the first semesters ^§B^ |
| **Dimension 5**  Antipsychotic drugs | Sustained low rates of use over time and /or decreasing use of antipsychotics over time : decreasing use of antipsychotic bitherapy, decreasing use of other antipsychotics in monotherapy and, to a lesser extent, decreasing use of risperidone were considered ^§B^  (particularly in the first years of follow-up) | Sustained limited rates of use of risperidone over time  Short and limited increasing use of risperidone over time  In clusters with concomitant high rates of psychosis comorbidity : sustained high use over time of antipsychotics other than risperidone | In clusters without concomitant high rates of psychosis comorbidity: sustained high rates of use and/or increasing use of antipsychotic bitherapy, or of antipsychotics other than risperidone |
| **Dimension 6**  Hospitalization for ADRD follow-up | Increasing rates of hospitalization for ADRD follow-up over time, whatever the length and type of hospital stay | Stable use over time | No use or decreasing use over time |
| **Dimension 7**  Hospitalization for ADRD complication or BPSD | Sustained low rates of hospitalization, particularly in the first years of follow-up | Slight progressive increasing use occurring in the last years of follow-up | High rate of use, and/or increasing use over time, particularly stays via the emergency rooms and during the first semesters of follow-up ^#^ |
| **Dimension 8**  Medical specialists ^$^ | Sustained use and/or increasing use of hospital specialist consultations (outpatient visit) ^µ^  To a lesser extent, sustained or increasing use of private specialists other than ADRD specialists | High rates of use when the cluster presented a high comorbidity profile | Decreasing use over time |
| **Dimension 9** Hospitalizations for causes other than ADRD (other than dimensions 6, 7 and 10) *^$^ | Sustained rates of use and/or increasing rates of use  ^$^ *, particularly for short-stay hospitalization suggesting planned outpatient stays for regular care such as cataract surgery | Increasing rates of long-stay hospitalizations in cluster presenting a high comorbidity profile  Increasing rates of short or long-stay hospitalizations in the semester before death | Decreased use over time , particularly for regular sessions due to comorbidities or decreasing use of short stay hospitalization suggesting planned outpatient stays for regular care such as cataract surgery ^$^ |
| **Dimension 10** Emergency | Limited baseline use, and no increasing use, in particular for emergency room without hospitalization over time | Slight increasing use of hospitalization in medicine or psychiatry after a stay in the emergency room, particularly in clusters presenting high levels of concomitant comorbidities | Increasing use over time of emergency room without hospitalization  High rate of use and/or huge increasing use particularly for clusters with low levels of concomitant comorbidities ^!^ |
| **Dimension 11** Institutionalization in a nursing home (NH) ^&^ | ~~-~~ | Progressive increasing rate of NH institutionalization over time, taking into account different expected rates across age class (with higher expected rates among the ≥85 yo)  Rapid increasing rate of institutionalization in clusters with high level of comorbidities involving dependency | Rapid and huge increased rate of early NH institutionalization in the first semesters, regardless of age class: institutionalization in this period appeared unfavourable, especially in the youngest (suggesting a potential late diagnosis and/or a rupture in the care pathway) |
| Death | Lower death rate and/or later death than in the whole age class | Death, whatever the time to death, among the ≥ 85 yo age class  Higher death rates than in the whole age class, whatever the time to death, among clusters with high life-threatening comorbidity profiles (e.g active cancer) | In the youngest age class (65-74), and at a lesser extent, in the 75-84 yo, in cluster without higher rates of concomitant life-threatening comorbidity : higher death rates occurring in the first semesters, suggesting potential late diagnosis and a previous erratic trajectory, particularly in the youngest age group |

*Abbreviations: ADRD: Alzheimer’s disease and related syndromes, NH : nursing home; yo : years old*

*^§A^ except during concomitant increasing high rates of institutionalization to a nursing home in the cluster (because during institutionalization in NH, all nursing care and some physiotherapist care delivered to NH residents are not captured in SNDS, due to the financing system of some of these structures)*

*^§B^ except during concomitant increasing high rates of institutionalization to a nursing home in the cluster: during institutionalization in NH with internal pharmacy, drugs reimbursements are not captured in SNDS data*

*^£^ In 2012, these drugs were still recommended*

*^#^ High rate of use, and/or Increasing use over time, particularly via the emergency rooms (category 7.2) during the first semesters of follow-up suggested a poor ambulatory healthcare, a possible insufficient follow-up for ADRD*

*^$^ Despite ADRD should not prevent healthcare use for various other conditions, literature showed that ADRD could involve an underuse of other specialists’ care, with less regular checkups for other comorbidities. Then, this was considered unfavourable, whereas a sustained use to another specialist was considered favourable.*

*^µ^ The hospital consultation specialty are not provided in the SDNS, therefore the category 8.1 covers all specialties. However, in France, most geriatricians work in hospitals, not in private settings: they were expected to contribute to part of the potential increase in the category 8.1*

** With increasing age, there is a mathematical increase in various hospitalizations causes, but ADRD may prevent this use for planned (non urgent) hospitalizations such as cataract surgery for example*

*^!^ High rate of use and/or huge increasing use of hospitalization in medicine or psychiatry after a stay in the emergency room may suggest previous underuse or low continuity of care for various diseases, particularly for clusters with low levels of concomitant comorbidities*

*^&^ Institutionalization in NH rates interpretation always took into account different expected rates across age class (with higher expected rates of NH institutionalization among the ≥85 yo than in the 75-84 yo, and than in the 65-74 yo)*

[1] Gallini A, Renoux A, Siep S, Roustan A, Voisin T, Escudier G, et al. Clustering 5-Year Multidimensional Health Care Trajectory Patterns in Alzheimer’s Disease and Related Syndromes. J Am Med Dir Assoc 2021;22:1525-1534.e3. https://doi.org/10.1016/j.jamda.2021.01.085.
