# Supplementary material for: Which factors preceding dementia identification impact future healthcare use trajectories: multilevel analyses in administrative data
Source: BMC Geriatr. 2024 Jan 23;24:89. doi: 10.1186/s12877-023-04643-1 (PMC10807194; doi:10.1186/s12877-023-04643-1)
Supplement: Supplementary file 2 — Additional file 2. Details on the construction and categorization of the healthcare use variables ([-18;-6] months). [file 12877_2023_4643_MOESM2_ESM.docx]

Additional file 2: Details on the construction and categorization of the healthcare use variables ([-18;-6] months)

| **Variable** | **Construction of the variable** | **Categorization** |
| --- | --- | --- |
| **Ambulatory setting** |  |  |
| General Practitioner’s (GP) consultations |  | None or once  Between two and four consultations  Between five and seven consultations  More than seven consultations |
| Ambulatory nursing care |  | None  Between once and four times  Five times and more |
| Physiotherapy sessions |  | None  Between one and ten sessions  Eleven sessions and more |
| Ambulatory cardiology consultation |  | None  At least once |
| Ambulatory surgery consultation |  | None  At least once |
| Ambulatory neurology consultation |  | None  At least once |
| Ambulatory psychiatry consultation |  | None  At least once |
| Ambulatory medical imaging |  | None  At least once |
| Ambulatory rheumatology/dermatology/ENT consultation | This variable gathered ambulatory dermatology, rheumatology and otorhinolaryngology (ear nose and throat (ENT)) consultations | None  At least once (for at least one of the three components) |
| Ambulatory other medical specialty consultation | This variable gathered ambulatory oncology, endocrinology, internal medicine and pulmonology consultations | None  At least once (for at least one of the four components) |
| Ambulatory visit to specialist with a frequent preventive approach (prevention consultation) | This variable gathered ambulatory dentist, gynecology and ophthalmology consultations | None  At least once (for at least one of the three components) |
| Preventive acts | This variable gathered flu vaccine and hearing test | None  At least once (for at least one of the two components) |
| Ambulatory allied health professional consultation | This variable gathered speech therapy, orthoptics and podiatry consultations | None  At least once (for at least one of the three components) |
| Outpatient consultation in hospital care |  | None  At least once |
| Medical transportation | This variable gathered ambulance and cab use | None  At least once (for one of the two components) |
| **Ambulatory drug exposure** |  |  |
| Antipsychotic | Number of packages  ATC code beginning with N05A | None  At least one |
| Antidepressant | Number of packages  ATC code beginning with N06A | None  At least one |
| Anxiolytic | Number of packages  ATC code beginning with N05BA or N05CD; or ATC code being N03AE01, M03BX07, N05BB01, N05BC01, N05BE01 | None  At least one |
| Z-drug | Number of packages  ATC code beginning with N05CF | None  At least one |
| Thymoregulator | Number of packages  ATC code being N05AN01, N03AG01, N03AG02 | None  At least one |
| Antalgic | Number of packages  ATC code beginning with N02 (excluding N02BE01) and ATC code being N03AX16 | None  At least one |
| Number of drugs | Number of packages  The number of drugs was accounted as the number of different substances (ATC code) during a quarter, excluding studied ATC classes (antipsychotic, antidepressant, anxiolytic, z-drug, thymoregulator, antalgic). Excessive polypharmacy was defined as using more than 10 different substances during a quarter. We used the highest value obtained during a quarter. [20] | No drug during the whole year  Between one and ten drugs during a quarter  Excessive polypharmacy |
| Number of potentially inappropriate medications (PIM) | EU(7)-PIM list [19] | No PIM  Between one and five PIM  Between six and ten PIM  Between eleven and 20 PIM  More than 20 PIM |
| **Medical devices** |  |  |
| Cane |  | None  At least once |
| Medical walker or wheelchair |  | None  At least once |
| Anti-bedsore cushion or mattress |  | None  At least once |
| Patient lift or medical bed |  | None  At least once |
| Nutritional supplement |  | None  At least once |
| **Hospitalization** |  |  |
| Short hospitalization | Hospitalization with the same entry and exit date, in any service (psychiatry, surgery, rehabilitation care) | None  Once  Twice and more |
| Cumulated duration of hospitalization stay(s) (>24h) | In this variable, the hospitalizations accounted were those with an exit date different from the entry date, in any service (psychiatry, surgery, rehabilitation care) | None  Between one and five days  Six days and more |
| Functional surgery | This variable gathered cataract, total hip replacement, total knee replacement. | None  At least once (for at least one of the three components) |
| Unplanned hospitalization | Hospitalization via the emergency room | None  At least once |
| Emergency room visit without hospitalization |  | None  At least once |
| Potentially Avoidable Hospitalization | Agency for Healthcare Research and Quality (AHRQ) Prevention Quality Indicators [17,18] | None  At least once |
| Hospitalization with neuropsychiatric disorder | ICD-10 codes of main or related diagnosis: F05; F06.2; F22; F23.3; F32-F39; F41.1-9; F51.2; F51.8; G47.0-2; G47.8-9; R40.0; R44; R45.0-5; R45.8; R46 | None  At least once |
| **Institutionalization** |  |  |
| Institutionalization | Drug exposure not measurable for institutionalized subjects in nursing home with an internal pharmacy [14] | No institutionalization  Nursing Home with an internal pharmacy  Nursing Home without an internal pharmacy |
