# Supplementary material for: Which factors preceding dementia identification impact future healthcare use trajectories: multilevel analyses in administrative data
Source: BMC Geriatr. 2024 Jan 23;24:89. doi: 10.1186/s12877-023-04643-1 (PMC10807194; doi:10.1186/s12877-023-04643-1)
Supplement: Supplementary file 3 — Additional file 3. Description of sociodemographics characteristics and healthcare use preceding ADRD identification ([-18;-6] months) by age group and according to the future favorable (or not) healthcare use trajectories (HUTs). [file 12877_2023_4643_MOESM3_ESM.docx]

Additional file 3: Description of sociodemographics characteristics and healthcare use before ADRD identification ([-18;-6] months) by age group and according to the favorable (or not) future healthcare use trajectory (HUT)

|  | **65-74 years group (n=4764)** | | | | | |  | **75-84 years group (n=16441)** | | | | | |  | **85 and older group (n=15785)** | | | | | |
| --- | --- | --- | --- | --- | --- | --- | --- | --- | --- | --- | --- | --- | --- | --- | --- | --- | --- | --- | --- | --- |
|  | **Unfavorable future HUT** | | **Favorable future HUT** | | **Total** | |  | **Unfavorable future HUT** | | **Favorable future HUT** | | **Total** | |  | **Unfavorable future HUT** | | **Favorable future HUT** | | **Total** | |
| **Sex** |  |  |  |  |  |  |  |  |  |  |  |  |  |  |  |  |  |  |  |  |
| Male | 1743 | (49.0%) | 477 | (39.6%) | 2220 | (46.6%) |  | 4639 | (36.7%) | 1187 | (31.3%) | 5826 | (35.4%) |  | 2648 | (23.4%) | 1035 | (23.3%) | 3683 | (23.3%) |
| Female | 1817 | (51.0%) | 727 | (60.4%) | 2544 | (53.4%) |  | 8008 | (63.3%) | 2607 | (68.7%) | 10615 | (64.6%) |  | 8691 | (76.6%) | 3411 | (76.7%) | 12102 | (76.7%) |
|  |  |  |  |  |  |  |  |  |  |  |  |  |  |  |  |  |  |  |  |  |
| **Age (continuous), mean (sd)** | 70.4 | (2.9) | 70.8 | (2.8) | 70.5 | (2.9) |  | 80.3 | (2.7) | 79.9 | (2.8) | 80.2 | (2.8) |  | 89.3 | (3.5) | 88.0 | (2.7) | 89.0 | (3.3) |
| **Number of comorbidities, mean (sd)** | 1.9 | (1.5) | 1.2 | (1.3) | 1.7 | (1.5) |  | 1.9 | (1.5) | 1.2 | (1.2) | 1.7 | (1.4) |  | 1.7 | (1.4) | 1.6 | (1.4) | 1.7 | (1.4) |
|  |  |  |  |  |  |  |  |  |  |  |  |  |  |  |  |  |  |  |  |  |
| **Type of location of residence** |  |  |  |  |  |  |  |  |  |  |  |  |  |  |  |  |  |  |  |  |
| Urban location | 2917 | (81.9%) | 969 | (80.5%) | 3886 | (81.6%) |  | 10539 | (83.3%) | 3160 | (83.3%) | 13699 | (83.3%) |  | 9640 | (85.0%) | 3795 | (85.4%) | 13435 | (85.1%) |
| Rural location | 643 | (18.1%) | 235 | (19.5%) | 878 | (18.4%) |  | 2108 | (16.7%) | 634 | (16.7%) | 2742 | (16.7%) |  | 1699 | (15.0%) | 651 | (14.6%) | 2350 | (14.9%) |
| **Deprivation index** |  |  |  |  |  |  |  |  |  |  |  |  |  |  |  |  |  |  |  |  |
| 1 | 619 | (17.4%) | 195 | (16.2%) | 814 | (17.1%) |  | 2179 | (17.2%) | 671 | (17.7%) | 2850 | (17.3%) |  | 2218 | (19.6%) | 963 | (21.7%) | 3181 | (20.2%) |
| 2 | 618 | (17.4%) | 243 | (20.2%) | 861 | (18.1%) |  | 2231 | (17.6%) | 752 | (19.8%) | 2983 | (18.1%) |  | 2107 | (18.6%) | 881 | (19.8%) | 2988 | (18.9%) |
| 3 | 694 | (19.5%) | 256 | (21.3%) | 950 | (19.9%) |  | 2529 | (20.0%) | 798 | (21.0%) | 3327 | (20.2%) |  | 2261 | (19.9%) | 888 | (20.0%) | 3149 | (19.9%) |
| 4 | 736 | (20.6%) | 236 | (19.6%) | 972 | (20.4%) |  | 2685 | (21.3%) | 766 | (20.2%) | 3451 | (21.00%) |  | 2282 | (20.1%) | 837 | (18.8%) | 3119 | (19.8%) |
| 5 (the most deprived) | 893 | (25.1%) | 274 | (22.7%) | 1167 | (24.5%) |  | 3023 | (23.9%) | 807 | (21.3%) | 3830 | (23.3%) |  | 2471 | (21.8%) | 877 | (19.7%) | 3348 | (21.2%) |
| **Institutionalization** |  |  |  |  |  |  |  |  |  |  |  |  |  |  |  |  |  |  |  |  |
| No institutionalization | 3406 | (95.7%) | 1200 | (99.7%) | 4606 | (96.7%) |  | 12041 | (95.2%) | 3783 | (99.7%) | 15824 | (96.3%) |  | 9854 | (86.9%) | 4280 | (96.2%) | 14134 | (89.5%) |
| Nursing home with internal pharmacy | 38 | (1.1%) | 0 | (0.00%) | 38 | (0.8%) |  | 136 | (1.1%) | 0 | (0.00%) | 136 | (0.8%) |  | 230 | (2.0%) | 16 | (0.4%) | 246 | (1.5%) |
| Nursing home without internal pharmacy | 116 | (3.2%) | 4 | (0.3%) | 120 | (2.5%) |  | 470 | (3.7%) | 11 | (0.3%) | 481 | (2.9%) |  | 1255 | (11.1%) | 150 | (3.4%) | 1405 | (8.9%) |
| **Number of General Practitioner consultations** |  |  |  |  |  |  |  |  |  |  |  |  |  |  |  |  |  |  |  |  |
| No or one GP consultation | 427 | (12.0%) | 91 | (7.6%) | 518 | (10.9%) |  | 1020 | (8.1%) | 212 | (5.6%) | 1232 | (7.5%) |  | 1047 | (9.2%) | 249 | (5.6%) | 1296 | (8.2%) |
| Between two and four GP consultations | 600 | (16.9%) | 252 | (20.9%) | 852 | (17.9%) |  | 1754 | (13.9%) | 618 | (16.3%) | 2372 | (14.4%) |  | 1590 | (14.1%) | 558 | (12.5%) | 2148 | (13.6%) |
| Between five and seven GP consultations | 824 | (23.1%) | 312 | (25.9%) | 1136 | (23.8%) |  | 2920 | (23.1%) | 963 | (25.4%) | 3883 | (23.6%) |  | 2431 | (21.4%) | 1034 | (23.3%) | 3465 | (22.0%) |
| More than seven GP consultations | 1709 | (48.0%) | 549 | (45.6%) | 2258 | (47.4%) |  | 6953 | (54.9%) | 2001 | (52.7%) | 8954 | (54.5%) |  | 6271 | (55.3%) | 2605 | (58.6%) | 8876 | (56.2%) |
| **Ambulatory nursing care** |  |  |  |  |  |  |  |  |  |  |  |  |  |  |  |  |  |  |  |  |
| None | 1628 | (45.7%) | 553 | (45.9%) | 2181 | (45.8%) |  | 4551 | (36.0%) | 1364 | (36.0%) | 5915 | (36.0%) |  | 4129 | (36.4%) | 1352 | (30.4%) | 5481 | (34.7%) |
| Between once and four times | 879 | (24.7%) | 362 | (30.1%) | 1241 | (26.1%) |  | 3391 | (26.8%) | 1188 | (31.3%) | 4579 | (27.8%) |  | 2595 | (22.9%) | 1188 | (26.7%) | 3783 | (24.0%) |
| Five times and more | 1053 | (29.6%) | 289 | (24.0%) | 1342 | (28.1%) |  | 4705 | (37.2%) | 1242 | (32.7%) | 5947 | (36.2%) |  | 4615 | (40.7%) | 1906 | (42.9%) | 6521 | (41.3%) |
|  |  |  |  |  |  |  |  |  |  |  |  |  |  |  |  |  |  |  |  |  |
| **Physiotherapy sessions** |  |  |  |  |  |  |  |  |  |  |  |  |  |  |  |  |  |  |  |  |
| None | 2558 | (71.8%) | 847 | (70.35%) | 3405 | (71.5%) |  | 8726 | (69.0%) | 2647 | (69.8%) | 11373 | (69.2%) |  | 7723 | (68.1%) | 2954 | (66.4%) | 10677 | (67.7%) |
| Between one and ten sessions | 252 | (7.1%) | 114 | (9.47%) | 366 | (7.7%) |  | 976 | (7.7%) | 348 | (9.2%) | 1324 | (8.0%) |  | 764 | (6.7%) | 360 | (8.1%) | 1124 | (7.1%) |
| 11 sessions and more | 750 | (21.1%) | 243 | (20.18%) | 993 | (20.8%) |  | 2945 | (23.3%) | 799 | (21.0%) | 3744 | (22.8%) |  | 2852 | (25.2%) | 1132 | (25.5%) | 3984 | (25.2%) |
| **Ambulatory cardiology consultation** |  |  |  |  |  |  |  |  |  |  |  |  |  |  |  |  |  |  |  |  |
| None | 2507 | (70.4%) | 835 | (69.4%) | 3342 | (70.2%) |  | 8537 | (67.5%) | 2534 | (66.8%) | 11071 | (67.3%) |  | 8532 | (75.2%) | 2926 | (65.8%) | 11458 | (72.6%) |
| At least once | 1053 | (29.6%) | 369 | (30.6%) | 1422 | (29.8%) |  | 4110 | (32.5%) | 1260 | (33.2%) | 5370 | (32.7%) |  | 2807 | (24.8%) | 1520 | (34.2%) | 4327 | (27.4%) |
| **Ambulatory surgery consultation** |  |  |  |  |  |  |  |  |  |  |  |  |  |  |  |  |  |  |  |  |
| None | 2756 | (77.4%) | 926 | (76.9%) | 3682 | (77.3%) |  | 9968 | (78.8%) | 2994 | (78.9%) | 12962 | (78.8%) |  | 9845 | (86.8%) | 3655 | (82.2%) | 13500 | (85.5%) |
| At least once | 804 | (22.6%) | 278 | (23.1%) | 1082 | (22.7%) |  | 2679 | (21.2%) | 800 | (21.1%) | 3479 | (21.2%) |  | 1494 | (13.2%) | 791 | (17.8%) | 2285 | (14.5%) |
| **Ambulatory psychiatry consultation** |  |  |  |  |  |  |  |  |  |  |  |  |  |  |  |  |  |  |  |  |
| None | 3260 | (91.6%) | 1121 | (93.1%) | 4381 | (92.0%) |  | 12224 | (96.7%) | 3670 | (96.7%) | 15894 | (96.7%) |  | 11201 | (98.8%) | 4364 | (98.2%) | 15565 | (98.6%) |
| At least once | 300 | (8.4%) | 83 | (6.9%) | 383 | (8.0%) |  | 423 | (3.3%) | 124 | (3.3%) | 547 | (3.3%) |  | 138 | (1.2%) | 82 | (1.8%) | 220 | (1.4%) |
| **Ambulatory neurology consultation** |  |  |  |  |  |  |  |  |  |  |  |  |  |  |  |  |  |  |  |  |
| None | 3447 | (96.8%) | 1170 | (97.2%) | 4617 | (96.9%) |  | 12434 | (98.3%) | 3720 | (98.1%) | 16154 | (98.3%) |  | 11288 | (99.5%) | 4405 | (99.1%) | 15693 | (99.4%) |
| At least once | 113 | (3.2%) | 34 | (2.8%) | 147 | (3.1%) |  | 213 | (1.7%) | 74 | (1.9%) | 287 | (1.7%) |  | 51 | (0.5%) | 41 | (0.9%) | 92 | (0.6%) |
| **Ambulatory dermatology/rheumatology/**  **otorhinolaryngology consultation** |  |  |  |  |  |  |  |  |  |  |  |  |  |  |  |  |  |  |  |  |
| None | 2693 | (75.6%) | 843 | (70.0%) | 3536 | (74.2%) |  | 9319 | (73.7%) | 2618 | (69.0%) | 11937 | (72.6%) |  | 8990 | (79.3%) | 3113 | (70.0%) | 12103 | (76.7%) |
| At least once | 867 | (24.4%) | 361 | (30.0%) | 1228 | (25.8%) |  | 3328 | (26.3%) | 1176 | (31.0%) | 4504 | (27.4%) |  | 2349 | (20.7%) | 1333 | (30.0%) | 3682 | (23.3%) |
| **Ambulatory other medical specialty consultation *** |  |  |  |  |  |  |  |  |  |  |  |  |  |  |  |  |  |  |  |  |
| None | 2626 | (73.8%) | 924 | (76.7%) | 3550 | (74.5%) |  | 10840 | (85.7%) | 3262 | (86.0%) | 14102 | (85.8%) |  | 10461 | (92.3%) | 3917 | (88.1%) | 14378 | (91.1%) |
| At least once | 934 | (26.2%) | 280 | (23.3%) | 1214 | (25.5%) |  | 1807 | (14.3%) | 532 | (14.0%) | 2339 | (14.2%) |  | 878 | (7.7%) | 529 | (11.9%) | 1407 | (8.9%) |
| **Ambulatory allied health professional consultation †** |  |  |  |  |  |  |  |  |  |  |  |  |  |  |  |  |  |  |  |  |
| None | 3195 | (89.7%) | 1064 | (88.37%) | 4259 | (89.40%) |  | 11592 | (91.66%) | 3457 | (91.12%) | 15049 | (91.53%) |  | 10747 | (94.78%) | 4112 | (92.49%) | 14859 | (94.13%) |
| At least once | 365 | (10.3%) | 140 | (11.63%) | 505 | (10.60%) |  | 1055 | (8.34%) | 337 | (8.88%) | 1392 | (8.47%) |  | 592 | (5.22%) | 334 | (7.51%) | 926 | (5.87%) |
| **Outpatient consultation in hospital care** |  |  |  |  |  |  |  |  |  |  |  |  |  |  |  |  |  |  |  |  |
| None | 1815 | (51.0%) | 690 | (57.3%) | 2505 | (52.6%) |  | 7306 | (57.8%) | 2316 | (61.0%) | 9622 | (58.5%) |  | 7538 | (66.5%) | 2836 | (63.8%) | 10374 | (65.7%) |
| At least once | 1745 | (49.0%) | 514 | (42.7%) | 2259 | (47.4%) |  | 5341 | (42.2%) | 1478 | (39.0%) | 6819 | (41.5%) |  | 3801 | (33.5%) | 1610 | (36.2%) | 5411 | (34.3%) |
| **Ambulatory prevention consultation ‡** |  |  |  |  |  |  |  |  |  |  |  |  |  |  |  |  |  |  |  |  |
| None | 1576 | (44.3%) | 447 | (37.1%) | 2023 | (42.5%) |  | 5677 | (44.9%) | 1352 | (35.6%) | 7029 | (42.7%) |  | 6301 | (55.6%) | 1885 | (42.4%) | 8186 | (51.9%) |
| At least once | 1984 | (55.7%) | 757 | (62.9%) | 2741 | (57.5%) |  | 6970 | (55.1%) | 2442 | (64.4%) | 9412 | (57.3%) |  | 5038 | (44.4%) | 2561 | (57.6%) | 7599 | (48.1%) |
|  |  |  |  |  |  |  |  |  |  |  |  |  |  |  |  |  |  |  |  |  |
| **Preventive act §** |  |  |  |  |  |  |  |  |  |  |  |  |  |  |  |  |  |  |  |  |
| None | 1903 | (53.5%) | 582 | (48.3%) | 2485 | (52.2%) |  | 5041 | (39.9%) | 1342 | (35.4%) | 6383 | (38.8%) |  | 4138 | (36.49%) | 1413 | (31.78%) | 5551 | (35.17%) |
| At least once | 1657 | (46.5%) | 622 | (51.7%) | 2279 | (47.8%) |  | 7606 | (60.1%) | 2452 | (64.6%) | 10058 | (61.2%) |  | 7201 | (63.51%) | 3033 | (68.22%) | 10234 | (64.83%) |
| **Ambulatory medical imaging** |  |  |  |  |  |  |  |  |  |  |  |  |  |  |  |  |  |  |  |  |
| None | 1551 | (43.6%) | 461 | (38.3%) | 2012 | (42.2%) |  | 6324 | (50.0%) | 1675 | (44.2%) | 7999 | (48.7%) |  | 7277 | (64.2%) | 2395 | (53.9%) | 9672 | (61.3%) |
| At least once | 2009 | (56.4%) | 743 | (61.7%) | 2752 | (57.8%) |  | 6323 | (50.0%) | 2119 | (55.8%) | 8442 | (51.3%) |  | 4062 | (35.8%) | 2051 | (46.1%) | 6113 | (38.7%) |
| **Cumulated duration of hospitalization stay(s)** |  |  |  |  |  |  |  |  |  |  |  |  |  |  |  |  |  |  |  |  |
| None | 2572 | (72.3%) | 991 | (82.3%) | 3563 | (74.8%) |  | 9611 | (76.0%) | 3150 | (83.0%) | 12761 | (77.6%) |  | 9168 | (80.8%) | 3579 | (80.5%) | 12747 | (80.8%) |
| Between one and five days | 278 | (7.8%) | 79 | (6.6%) | 357 | (7.5%) |  | 917 | (7.3%) | 261 | (6.9%) | 1178 | (7.2%) |  | 460 | (4.1%) | 225 | (5.1%) | 685 | (4.3%) |
| Six days and more | 710 | (19.9%) | 134 | (11.1%) | 844 | (17.7%) |  | 2119 | (16.7%) | 383 | (10.1%) | 2502 | (15.2%) |  | 1711 | (15.1%) | 642 | (14.4%) | 2353 | (14.9%) |
| **Short hospitalization (same entry and exit date)** |  |  |  |  |  |  |  |  |  |  |  |  |  |  |  |  |  |  |  |  |
| None | 2960 | (83.1%) | 999 | (83.0%) | 3959 | (83.1%) |  | 10976 | (86.8%) | 3244 | (85.5%) | 14220 | (86.5%) |  | 10625 | (93.7%) | 4015 | (90.3%) | 14640 | (92.8%) |
| Once | 377 | (10.6%) | 146 | (12.1%) | 523 | (11.0%) |  | 1070 | (8.5%) | 373 | (9.8%) | 1443 | (8.8%) |  | 488 | (4.3%) | 308 | (6.9%) | 796 | (5.0%) |
| At least twice | 223 | (6.3%) | 59 | (4.9%) | 282 | (5.9%) |  | 601 | (4.7%) | 177 | (4.7%) | 778 | (4.7%) |  | 226 | (2.00%) | 123 | (2.8%) | 349 | (2.2%) |
| **Emergency room visit without hospitalization** |  |  |  |  |  |  |  |  |  |  |  |  |  |  |  |  |  |  |  |  |
| None | 2995 | (84.1%) | 1081 | (89.8%) | 4076 | (85.6%) |  | 10830 | (85.6%) | 3413 | (90.0%) | 14243 | (86.6%) |  | 9700 | (85.5%) | 3861 | (86.8%) | 13561 | (85.9%) |
| At least once | 565 | (15.9%) | 123 | (10.2%) | 688 | (14.4%) |  | 1817 | (14.4%) | 381 | (10.0%) | 2198 | (13.4%) |  | 1639 | (14.5%) | 585 | (13.2%) | 2224 | (14.1%) |
| **Unplanned hospitalization (via the emergency room)** |  |  |  |  |  |  |  |  |  |  |  |  |  |  |  |  |  |  |  |  |
| None | 2835 | (79.6%) | 1059 | (88.0%) | 3894 | (81.7%) |  | 10106 | (79.9%) | 3374 | (88.9%) | 13480 | (82.0%) |  | 8848 | (78.0%) | 3598 | (80.9%) | 12446 | (78.9%) |
| At least once | 725 | (20.4%) | 145 | (12.0%) | 870 | (18.3%) |  | 2541 | (20.1%) | 420 | (11.1%) | 2961 | (18.0%) |  | 2491 | (22.0%) | 848 | (19.1%) | 3339 | (21.1%) |
| **Potentially Avoidable Hospitalization** |  |  |  |  |  |  |  |  |  |  |  |  |  |  |  |  |  |  |  |  |
| None | 3427 | (96.3%) | 1184 | (98.3%) | 4611 | (96.8%) |  | 12138 | (96.0%) | 3729 | (98.3%) | 15867 | (96.5%) |  | 10793 | (95.2%) | 4268 | (96.0%) | 15061 | (95.4%) |
| At least once | 133 | (3.7%) | 20 | (1.7%) | 153 | (3.2%) |  | 509 | (4.0%) | 65 | (1.7%) | 574 | (3.5%) |  | 546 | (4.8%) | 178 | (4.0%) | 724 | (4.6%) |
| **Hospitalization with neuropsychiatric disorder** |  |  |  |  |  |  |  |  |  |  |  |  |  |  |  |  |  |  |  |  |
| None | 3407 | (95.7%) | 1181 | (98.1%) | 4588 | (96.3%) |  | 12339 | (97.6%) | 3747 | (98.8%) | 16086 | (97.8%) |  | 11151 | (98.3%) | 4389 | (98.7%) | 15540 | (98.4%) |
| At least once | 153 | (4.3%) | 23 | (1.9%) | 176 | (3.7%) |  | 308 | (2.4%) | 47 | (1.2%) | 355 | (2.2%) |  | 188 | (1.7%) | 57 | (1.3%) | 245 | (1.6%) |
| **Functional surgery ¶** |  |  |  |  |  |  |  |  |  |  |  |  |  |  |  |  |  |  |  |  |
| None | 3440 | (96.6%) | 1157 | (96.1%) | 4597 | (96.5%) |  | 12029 | (95.1%) | 3601 | (94.9%) | 15630 | (95.1%) |  | 11033 | (97.3%) | 4267 | (96.0%) | 15300 | (96.9%) |
| At least once | 120 | (3.4%) | 47 | (3.9%) | 167 | (3.5%) |  | 618 | (4.9%) | 193 | (5.1%) | 811 | (4.9%) |  | 306 | (2.7%) | 179 | (4.0%) | 485 | (3.1%) |
|  |  |  |  |  |  |  |  |  |  |  |  |  |  |  |  |  |  |  |  |  |
|  |  |  |  |  |  |  |  |  |  |  |  |  |  |  |  |  |  |  |  |  |
|  |  |  |  |  |  |  |  |  |  |  |  |  |  |  |  |  |  |  |  |  |
| **Number of drugs #** |  |  |  |  |  |  |  |  |  |  |  |  |  |  |  |  |  |  |  |  |
| No drug (year) | 267 | (7.5%) | 52 | (4.3%) | 319 | (6.7%) |  | 624 | (4.9%) | 100 | (2.6%) | 724 | (4.4%) |  | 689 | (6.1%) | 122 | (2.7%) | 811 | (5.1%) |
| Between one and nine drugs (quarter) | 2179 | (61.2%) | 868 | (72.1%) | 3047 | (64.0%) |  | 7788 | (61.6%) | 2585 | (68.1%) | 10373 | (63.1%) |  | 7547 | (66.6%) | 2806 | (63.1%) | 10353 | (65.6%) |
| Excessive polypharmacy (quarter) | 1114 | (31.3%) | 284 | (23.6%) | 1398 | (29.3%) |  | 4235 | (33.5%) | 1109 | (29.3%) | 5344 | (32.5%) |  | 3103 | (27.3%) | 1518 | (34.2%) | 4621 | (29.3%) |
| **Number of PIM #** |  |  |  |  |  |  |  |  |  |  |  |  |  |  |  |  |  |  |  |  |
| No PIM | 547 | (15.4%) | 194 | (16.1%) | 741 | (15.6%) |  | 1730 | (13.7%) | 451 | (11.9%) | 2181 | (13.3%) |  | 1712 | (15.1%) | 476 | (10.7%) | 2188 | (13.9%) |
| Between one and five PIM | 1789 | (50.3%) | 674 | (56.0%) | 2463 | (51.7%) |  | 6773 | (53.5%) | 2265 | (59.7%) | 9038 | (54.9%) |  | 6255 | (55.2%) | 2483 | (55.8%) | 8738 | (55.4%) |
| Between six and ten PIM | 553 | (15.5%) | 162 | (13.5%) | 715 | (15.0%) |  | 1632 | (12.9%) | 486 | (12.8%) | 2118 | (12.9%) |  | 1149 | (10.1%) | 572 | (12.9%) | 1721 | (10.9%) |
| Between 11 and 20 PIM | 400 | (11.2%) | 116 | (9.6%) | 516 | (10.8%) |  | 1438 | (11.4%) | 354 | (9.3%) | 1792 | (10.9%) |  | 1411 | (12.4%) | 541 | (12.2%) | 1952 | (12.4%) |
| More than 20 PIM | 271 | (7.6%) | 58 | (4.8%) | 329 | (6.9%) |  | 1074 | (8.5%) | 238 | (6.3%) | 1312 | (8.0%) |  | 812 | (7.2%) | 374 | (8.4%) | 1186 | (7.4%) |
| **Antipsychotic** |  |  |  |  |  |  |  |  |  |  |  |  |  |  |  |  |  |  |  |  |
| None | 3048 | (85.6%) | 1129 | (93.8%) | 4177 | (87.7%) |  | 11616 | (91.9%) | 3614 | (95.3%) | 15230 | (92.6%) |  | 10505 | (92.6%) | 4202 | (94.5%) | 14707 | (93.2%) |
| At least once | 512 | (14.4%) | 75 | (6.2%) | 587 | (12.3%) |  | 1031 | (8.1%) | 180 | (4.7%) | 1211 | (7.4%) |  | 834 | (7.4%) | 244 | (5.5%) | 1078 | (6.8%) |
| **Antidepressant** |  |  |  |  |  |  |  |  |  |  |  |  |  |  |  |  |  |  |  |  |
| None | 2240 | (62.9%) | 746 | (62.0%) | 2986 | (62.7%) |  | 8337 | (65.9%) | 2406 | (63.4%) | 10743 | (65.3%) |  | 7836 | (69.1%) | 2985 | (67.1%) | 10821 | (68.5%) |
| At least once | 1320 | (37.1%) | 458 | (38.0%) | 1778 | (37.3%) |  | 4310 | (34.1%) | 1388 | (36.6%) | 5698 | (34.7%) |  | 3503 | (30.9%) | 1461 | (32.9%) | 4964 | (31.5%) |
| **Z-drug** |  |  |  |  |  |  |  |  |  |  |  |  |  |  |  |  |  |  |  |  |
| None | 2835 | (79.6%) | 994 | (82.6%) | 3829 | (80.4%) |  | 10192 | (80.6%) | 3101 | (81.7%) | 13293 | (80.8%) |  | 9294 | (82.0%) | 3533 | (79.5%) | 12827 | (81.3%) |
| At least once | 725 | (20.4%) | 210 | (17.4%) | 935 | (19.6%) |  | 2455 | (19.4%) | 693 | (18.3%) | 3148 | (19.2%) |  | 2045 | (18.0%) | 913 | (20.5%) | 2958 | (18.7%) |
| **Anxiolytic** |  |  |  |  |  |  |  |  |  |  |  |  |  |  |  |  |  |  |  |  |
| None | 1951 | (54.8%) | 735 | (61.0%) | 2686 | (56.4%) |  | 7380 | (58.3%) | 2270 | (59.8%) | 9650 | (58.7%) |  | 6912 | (61.0%) | 2592 | (58.3%) | 9504 | (60.2%) |
| At least once | 1609 | (45.2%) | 469 | (39.0%) | 2078 | (43.6%) |  | 5267 | (41.7%) | 1524 | (40.2%) | 6791 | (41.3%) |  | 4427 | (39.0%) | 1854 | (41.7%) | 6281 | (39.8%) |
| **Thymoregulator** |  |  |  |  |  |  |  |  |  |  |  |  |  |  |  |  |  |  |  |  |
| None | 3425 | (96.2%) | 1168 | (97.0%) | 4593 | (96.4%) |  | 12457 | (98.5%) | 3766 | (99.3%) | 16223 | (98.7%) |  | 11292 | (99.6%) | 4424 | (99.5%) | 15716 | (99.6%) |
| At least once | 135 | (3.8%) | 36 | (3.0%) | 171 | (3.6%) |  | 190 | (1.5%) | 28 | (0.7%) | 218 | (1.3%) |  | 47 | (0.4%) | 22 | (0.5%) | 69 | (0.4%) |
| **Antalgic** |  |  |  |  |  |  |  |  |  |  |  |  |  |  |  |  |  |  |  |  |
| None | 2326 | (65.3%) | 818 | (67.9%) | 3144 | (66.0%) |  | 8118 | (64.2%) | 2499 | (65.9%) | 10617 | (64.6%) |  | 7848 | (69.2%) | 2813 | (63.3%) | 10661 | (67.5%) |
| At least once | 1234 | (34.7%) | 386 | (32.1%) | 1620 | (34.0%) |  | 4529 | (35.8%) | 1295 | (34.1%) | 5824 | (35.4%) |  | 3491 | (30.8%) | 1633 | (36.7%) | 5124 | (32.5%) |
| **Medical transportation (ambulance or cab)** |  |  |  |  |  |  |  |  |  |  |  |  |  |  |  |  |  |  |  |  |
| None | 2289 | (64.3%) | 951 | (79.0%) | 3240 | (68.0%) |  | 8035 | (63.5%) | 2929 | (77.2%) | 10964 | (66.7%) |  | 7094 | (62.6%) | 2978 | (67.0%) | 10072 | (63.8%) |
| At least once | 1271 | (35.7%) | 253 | (21.0%) | 1524 | (32.0%) |  | 4612 | (36.5%) | 865 | (22.8%) | 5477 | (33.3%) |  | 4245 | (37.4%) | 1468 | (33.0%) | 5713 | (36.2%) |
|  |  |  |  |  |  |  |  |  |  |  |  |  |  |  |  |  |  |  |  |  |
| **Cane** |  |  |  |  |  |  |  |  |  |  |  |  |  |  |  |  |  |  |  |  |
| No | 3424 | (96.2%) | 1164 | (96.7%) | 4588 | (96.3%) |  | 12115 | (95.8%) | 3671 | (96.8%) | 15786 | (96.0%) |  | 10959 | (96.7%) | 4248 | (95.5%) | 15207 | (96.3%) |
| Yes | 136 | (3.8%) | 40 | (3.3%) | 176 | (3.7%) |  | 532 | (4.2%) | 123 | (3.2%) | 655 | (4.0%) |  | 380 | (3.3%) | 198 | (4.5%) | 578 | (3.7%) |
| **Medical walker or wheelchair** |  |  |  |  |  |  |  |  |  |  |  |  |  |  |  |  |  |  |  |  |
| No | 3364 | (94.5%) | 1159 | (96.3%) | 4523 | (94.9%) |  | 11822 | (93.5%) | 3679 | (97.0%) | 15501 | (94.3%) |  | 10276 | (90.6%) | 4098 | (92.2%) | 14374 | (91.1%) |
| Yes | 196 | (5.5%) | 45 | (3.7%) | 241 | (5.1%) |  | 825 | (6.5%) | 115 | (3.0%) | 940 | (5.7%) |  | 1063 | (9.4%) | 348 | (7.8%) | 1411 | (8.9%) |
| **Anti-bedsore cushion or mattress** |  |  |  |  |  |  |  |  |  |  |  |  |  |  |  |  |  |  |  |  |
| No | 3433 | (96.4%) | 1178 | (97.8%) | 4611 | (96.8%) |  | 12129 | (95.9%) | 3708 | (97.7%) | 15837 | (96.3%) |  | 10734 | (94.7%) | 4258 | (95.8%) | 14992 | (95.0%) |
| Yes | 127 | (3.6%) | 26 | (2.2%) | 153 | (3.2%) |  | 518 | (4.1%) | 86 | (2.3%) | 604 | (3.7%) |  | 605 | (5.3%) | 188 | (4.2%) | 793 | (5.0%) |
| **Patient lift or medical bed** |  |  |  |  |  |  |  |  |  |  |  |  |  |  |  |  |  |  |  |  |
| No | 3414 | (95.9%) | 1162 | (96.5%) | 4576 | (96.0%) |  | 11878 | (93.9%) | 3678 | (96.9%) | 15556 | (94.6%) |  | 10366 | (91.4%) | 4174 | (93.9%) | 14540 | (92.1%) |
| Yes | 146 | (4.1%) | 42 | (3.5%) | 188 | (4.0%) |  | 769 | (6.1%) | 116 | (3.1%) | 885 | (5.4%) |  | 973 | (8.6%) | 272 | (6.1%) | 1245 | (7.9%) |
| **Nutritional supplement** |  |  |  |  |  |  |  |  |  |  |  |  |  |  |  |  |  |  |  |  |
| None | 3347 | (94.0%) | 1159 | (96.3%) | 4506 | (94.6%) |  | 11653 | (92.1%) | 3589 | (94.6%) | 15242 | (92.7%) |  | 9832 | (86.7%) | 3974 | (89.4%) | 13806 | (87.5%) |
| At least once | 213 | (6.0%) | 45 | (3.7%) | 258 | (5.4%) |  | 994 | (7.9%) | 205 | (5.4%) | 1199 | (7.3%) |  | 1507 | (13.3%) | 472 | (10.6%) | 1979 | (12.5%) |
| *HUT: Healthcare Use Trajectory; sd: standard deviation; GP: General Practitioner; PIM: Potentially Inappropriate Medication*  **ambulatory oncology, endocrinology, internal medicine, pulmonology consultations*  **†** *speech therapy, orthoptics, podiatry consultations*  **‡** *ambulatory dentist, gynecology, ophthalmology consultations*  **§** *flu vaccine, hearing test*  **¶** *cataract, total hip replacement, total knee replacement*  *# excluding antipsychotic, antidepressant, anxiolytic, z-drug, thymoregulator, antalgic* | | | | | | | | | | | | | | | | | | | | |
