# Supplementary material for: Which factors preceding dementia identification impact future healthcare use trajectories: multilevel analyses in administrative data
Source: BMC Geriatr. 2024 Jan 23;24:89. doi: 10.1186/s12877-023-04643-1 (PMC10807194; doi:10.1186/s12877-023-04643-1)
Supplement: Supplementary file 4 — Additional file 4. Results of the multilevel multivariate analysis of factors associated with future favorable healthcare use trajectories of the 65-74 years group (n=4,764). [file 12877_2023_4643_MOESM4_ESM.docx]

# Additional file 4: Results of the multilevel multivariate analysis of factors associated with a favorable future healthcare use trajectory of the 65-74 years group (n=4,764)

|  | **65-74 years group** | | | | | | |
| --- | --- | --- | --- | --- | --- | --- | --- |
|  | **(n=4,764)** | | | | | | |
|  | **Bivariate analysis** | | |  | **Multivariate analysis** | | |
|  | **OR** | **95%CI** | **p-value** |  | **aOR** | **95%CI** | **p-value** |
| **Sociodemographic caracteristics** |  |  |  |  |  |  |  |
| **Sex** |  |  |  |  |  |  |  |
| Female | 1.48 | [1.29-1.69] | < .001 *** |  | 1.34 | [1.16-1.54] | < .001 *** |
| **Age (continuous)** | 1.05 | [1.02-1.07] | < .001 *** |  | 1.03 | [1.00-1.05] | 0.03 * |
| **Number of comorbidities (continuous)** | 0.66 | [0.62-0.70] | < .001 *** |  | 0.70 | [0.66-0.75] | < .001 *** |
| **Type of location of residence** |  |  |  |  |  |  |  |
| Rural location | 1.072 | [0.901-1.275] | 0.431 NS |  |  |  |  |
| **Deprivation index** |  |  |  |  |  |  |  |
| 2 | 1.074 | [0.844-1.366] | 0.194 NS |  |  |  |  |
| 3 | 0.993 | [0.778-1.263] |  |  |  |  |  |
| 4 | 0.878 | [0.689-1.120] |  |  |  |  |  |
| 5 (the most deprived) | 0.850 | [0.670-1.078] |  |  |  |  |  |
| **Healthcare use before ADRD identification** |  |  |  |  |  |  |  |
| **Institutionalization** |  |  |  |  |  |  |  |
| Nursing home with internal pharmacy | 9.80E-08 | [6.95E-09 - 1.38E-06] | < .001 *** |  | 1.98E-07 | [1.22E-08 - 3.21E-06] | < .001 *** |
| Nursing home without internal pharmacy | 0.09 | [0.03-0.25] |  |  | 0.152 | [0.06-0.42] |  |
| **General Practicioner** |  |  |  |  |  |  |  |
| No or one GP consultation | 0.52 | [0.39-0.68] | < .001 *** |  |  |  |  |
| Between five and seven GP consultations | 0.88 | [0.72-1.08] |  |  |  |  |  |
| More than seven consultations | 0.73 | [0.61-0.87] |  |  |  |  |  |
| **Ambulatory nursing care** |  |  |  |  |  |  |  |
| Between once and four times | 1.19 | [1.02-1.40] | < .001 *** |  |  |  |  |
| Five times and more | 0.78 | [0.66-0.92] |  |  |  |  |  |
|  |  |  |  |  |  |  |  |
|  |  |  |  |  |  |  |  |
| **Physiotherapy sessions** |  |  |  |  |  |  |  |
| Between one and ten sessions | 1.37 | [1.08-1.73] | 0.03 * |  |  |  |  |
| More than ten sessions | 0.96 | [0.81-1.14] |  |  |  |  |  |
| **Ambulatory cardiology consultation (at least once)** | 1.04 | [0.90-1.20] | 0.62 |  | 1.25 | [1.07-1.47] | < .01 ** |
| **Ambulatory surgery consultation (at least once)** | 1.04 | [0.89-1.22] | 0.64 |  |  |  |  |
| **Ambulatory psychiatry consultation (at least once)** | 0.82 | [0.64-1.06] | 0.13 |  |  |  |  |
| **Ambulatory neurology consultation (at least once)** | 0.87 | [0.59-1.29] | 0.48 |  |  |  |  |
| **Ambulatory dermatology/rheumatology/otorhinolaryngology consultation (at least once)** | 1.34 | [1.16-1.56] | < .001 *** |  |  |  |  |
| **Ambulatory other medical specialty consultation (at least once) *** | 0.87 | [0.74-1.01] | 0.08 |  |  |  |  |
| **Ambulatory allied health professional consultation (at least once) †** | 1.16 | [0.94-1.43] | 0.17 |  |  |  |  |
| **Outpatient consultation in hospital care (at least once)** | 0.80 | [0.70-0.91] | < .01 ** |  |  |  |  |
| **Prevention consultation (at least once) ‡** | 1.35 | [1.18-1.54] | < .001 *** |  |  |  |  |
| **Preventive act (at least once) §** | 1.21 | [1.06-1.38] | < .01 ** |  | 1.23 | [1.06-1.42] | < .01 ** |
| **Ambulatory medical imaging (at least once)** | 1.25 | [1.09-1.43] | < .01 ** |  | 1.18 | [1.02-1.37] | 0.03 * |
| **Cumulated duration of planned hospitalization stay(s)** |  |  |  |  |  |  |  |
| Between one and five days | 0.74 | [0.57-0.96] | < .001 *** |  |  |  |  |
| More than five days | 0.50 | [0.41-0.61] |  |  |  |  |  |
| **Planned short hospitalization (same entry and exit date)** |  |  |  |  |  |  |  |
| Once | 1.16 | [0.94-1.43] | 0.10 |  |  |  |  |
| At least twice | 0.80 | [0.59-1.07] |  |  |  |  |  |
| **Emergency room visit without hospitalization (at least once)** | 0.62 | [0.50-0.76] | < .001 *** |  |  |  |  |
| **Unplanned hospitalization (via the emergency room) (at least once)** | 0.54 | [0.44-0.65] | < .001 *** |  |  |  |  |
| **Potentially avoidable hospitalization (at least once)** | 0.44 | [0.27-0.71] | < .01 ** |  |  |  |  |
| **Hospitalization with neuropsychiatric disorder (at least once)** | 0.44 | [0.28-0.68] | < .001 *** |  |  |  |  |
| **Functional surgery (at least once) ¶** | 1.15 | [0.81-1.62] | 0.44 |  |  |  |  |
| **Antipsychotic (at least once)** | 0.39 | [0.30-0.50] | < .001 *** |  | 0.57 | [0.43-0.74] | < .001 *** |
| **Antidepressant (at least once)** | 1.04 | [0.91-1.20] | 0.55 |  | 1.30 | [1.11-1.53] | < .01 ** |
| **Anxiolytic (at least once)** | 0.77 | [0.67-0.88] | < .001 *** |  | 0.85 | [0.72-0.99] | 0.04 * |
| **Z-drug (at least once)** | 0.83 | [0.70-0.98] | 0.03 * |  |  |  |  |
| **Antalgic (at least once)** | 0.90 | [0.78-1.03] | 0.13 |  |  |  |  |
| **Thymoregulator (at least once)** | 0.77 | [0.53-1.12] | 0.16 |  |  |  |  |
|  |  |  |  |  |  |  |  |
| **Number of drugs #** |  |  |  |  |  |  |  |
| No drug (year) | 0.49 | [0.36-0.67] | < .001 *** |  | 0.60 | [0.43-0.84] | < .01 ** |
| Excessive polypharmacy (quarter) | 0.63 | [0.54-0.74] |  |  | 0.86 | [0.72-1.02] |  |
| **Number of PIM #** |  |  |  |  |  |  |  |
| Between one and five PIM | 1.00 | [0.85-1.18] | 0.03 * |  |  |  |  |
| Between six and ten PIM | 0.94 | [0.71-1.25] |  |  |  |  |  |
| Between 11 and 20 PIM | 0.84 | [0.64-1.09] |  |  |  |  |  |
| More than 20 PIM | 0.56 | [0.38-0.84] |  |  |  |  |  |
| **Medical transportation (at least once)** | 0.47 | [0.41-0.55] | < .001 *** |  | 0.73 | [0.61-0.87] | < .01 ** |
| **Cane** | 0.88 | [0.61-1.27] | 0.50 |  |  |  |  |
| **Medical walker of wheelchair** | 0.67 | [0.48-0.93] | 0.02 * |  |  |  |  |
| **Anti-bedsore cushion or mattress** | 0.59 | [0.38-0.90] | 0.02 * |  |  |  |  |
| **Patient lift or medical bed** | 0.83 | [0.59-1.19] | 0.32 |  |  |  |  |
| **Nutritional supplement** | 0.62 | [0.44-0.86] | < .01 ** |  |  |  |  |
|  |  |  |  |  |  |  |  |
| **Interdepartmental variance** | 0.08 | [0.04-0.16] | < .001 *** |  | 0.07 | [0.03-0.15] | < .001 *** |
| *PIM: Potentially Inappropriate Medication*  **ambulatory oncology, endocrinology, internal medicine, pulmonology consultations*  **†** *speech therapy, orthoptics, podiatry consultations*  **‡** *ambulatory dentist, gynecology, ophthalmology consultations*  **§** *flu vaccine, hearing test*  **¶** *cataract, total hip replacement, total knee replacement*  *# excluding antipsychotic, antidepressant, anxiolytic, z-drug, thymoregulator, antalgic* |  |  |  |  |  |  |  |
