# Supplementary material for: Which factors preceding dementia identification impact future healthcare use trajectories: multilevel analyses in administrative data
Source: BMC Geriatr. 2024 Jan 23;24:89. doi: 10.1186/s12877-023-04643-1 (PMC10807194; doi:10.1186/s12877-023-04643-1)
Supplement: Supplementary file 5 — Additional file 5. Results of the multilevel multivariate analysis of factors associated with future favorable healthcare use trajectories of the 75-84 years group (n=16,441). [file 12877_2023_4643_MOESM5_ESM.docx]

# Additional file 5: Results of the multilevel multivariate analysis of factors associated with a favorable future healthcare use trajectory of the 75-84 years group (n=16,441)

|  | **75-84 years group** | | | | | | |  |
| --- | --- | --- | --- | --- | --- | --- | --- | --- |
|  | **(n=16,441)** | | | | | | |  |
|  | **Bivariate analysis** | | |  | **Multivariate analysis** | | | |
|  | **OR** | **IC95%** | **p-value** |  | **aOR** | **IC95%** | **p-value** | |
| **Sociodemographic caracteristics** |  |  |  |  |  |  |  | |
| **Sex** |  |  |  |  |  |  |  | |
| Female | 1.28 | [1.18-1.38] | < .001*** |  | 1.15 | [1.06-1.25] | < .01 ** | |
| **Age (continuous)** | 0.95 | [0.94-0.96] | < .001 *** |  | 0.95 | [0.94-0.96] | < .001 *** | |
| **Number of comorbidities (continuous)** | 0.69 | [0.66-0.71] | < .001 *** |  | 0.71 | [0.68-0.73] | < .001 *** | |
| **Type of location of residence** |  |  |  |  |  |  |  | |
| Rural location | 1.05 | [0.94-1.16] | 0.39 |  |  |  |  | |
| **Deprivation index** |  |  |  |  |  |  |  | |
| 2 | 0.93 | [0.81-1.06] | < .001 *** |  |  |  |  | |
| 3 | 0.85 | [0.75-0.98] |  |  |  |  |  | |
| 4 | 0.78 | [0.68-0.89] |  |  |  |  |  | |
| 5 (the most deprived) | 0.76 | [0.67-0.88] |  |  |  |  |  | |
| **Healthcare use before ADRD identification** |  |  |  |  |  |  |  | |
| **Institutionalization** |  |  |  |  |  |  |  | |
| Nursing home with internal pharmacy | 1.85E-07 | [4.66E-08 - 7.31E-07] | < .001 *** |  | 3.72E-07 | [9.15E-08 - 1.51E-06] | < .001 *** | |
| Nursing home without internal pharmacy | 0.07 | [0.04-0.13] |  |  | 0.11 | [0.06-0.19] |  |  |
| **General Practicioner** |  |  |  |  |  |  |  | |
| No or one GP consultation | 0.58 | [0.48-0.69] | < .001 *** |  |  |  |  | |
| Between five and seven GP consultations | 0.92 | [0.82-1.04] |  |  |  |  |  | |
| More than seven consultations | 0.78 | [0.70-0.86] |  |  |  |  |  | |
| **Ambulatory nursing care** |  |  |  |  |  |  |  | |
| Between once and four times | 1.20 | [1.10-1.32] | < .001 *** |  |  |  |  | |
| Five times and more | 0.85 | [0.78-0.93] |  |  |  |  |  | |
| **Physiotherapy sessions** |  |  |  |  |  |  |  | |
| Between one and ten sessions | 1.18 | [1.03-1.34] | < .001 *** |  | 1.23 | [1.07-1.41] | 0.01 * | |
| More than ten sessions | 0.85 | [0.77-0.93] |  |  | 1.07 | [0.96-1.18] |  |  |
| **Ambulatory cardiology consultation (at least once)** | 1.01 | [0.93-1.09] | 0.83 |  | 1.13 | [1.03-1.23] | < .01 ** | |
| **Ambulatory surgery consultation (at least once)** | 0.99 | [0.90-1.08] | 0.74 |  |  |  |  | |
| **Ambulatory psychiatry consultation (at least once)** | 0.98 | [0.79-1.20] | 0.82 |  |  |  |  | |
| **Ambulatory neurology consultation (at least once)** | 1.15 | [0.87-1.50] | 0.33 |  |  |  |  | |
| **Ambulatory dermatology/rheumatology/otorhinolaryngology consultation (at least once)** | 1.25 | [1.15-1.35] | < .001 *** |  |  |  |  | |
| **Ambulatory other medical specialty consultation (at least once) *** | 0.97 | [0.87-1.07] | 0.52 |  |  |  |  | |
| **Ambulatory allied health professional consultation (at least once) †** | 1.04 | [0.91-1.19] | 0.55 |  |  |  |  | |
| **Outpatient consultation in hospital care (at least once)** | 0.91 | [0.85-0.98] | 0.02 * |  | 1.14 | [1.04-1.25] | < .01 ** | |
| **Prevention consultation (at least once) ‡** | 1.47 | [1.36-1.59] | < .001 *** |  | 1.26 | [1.16-1.37] | < .001 *** | |
| **Preventive act (at least once) §** | 1.22 | [1.13-1.31] | < .001 *** |  | 1.18 | [1.08-1.28] | < .001 *** | |
| **Ambulatory medical imaging (at least once)** | 1.25 | [1.16-1.35] | < .001 *** |  | 1.19 | [1.10-1.30] | < .001 *** | |
| **Cumulated duration of planned hospitalization stay(s)** |  |  |  |  |  |  |  | |
| Between one and five days | 0.87 | [0.75-1.00] | < .001 *** |  |  |  |  | |
| More than five days | 0.54 | [0.48-0.61] |  |  |  |  |  | |
| **Planned short hospitalization (same entry and exit date)** |  |  |  |  |  |  |  | |
| Once | 1.16 | [1.02-1.31] | 0.07 |  |  |  |  | |
| At least twice | 1.00 | [0.84-1.19] |  |  |  |  |  | |
| **Emergency room visit without hospitalization (at least once)** | 0.69 | [0.61-0.77] | < .001 *** |  | 0.77 | [0.67-0.88] | < .001 *** | |
| **Unplanned hospitalization (via the emergency room) (at least once)** | 0.49 | [0.44-0.55] | < .001 *** |  | 0.81 | [0.71-0.92] | < .01 ** | |
| **Potentially avoidable hospitalization (at least once)** | 0.42 | [0.32-0.54] | < .001 *** |  |  |  |  | |
| **Hospitalization with neuropsychiatric disorder (at least once)** | 0.51 | [0.38-0.70] | < .001 *** |  |  |  |  | |
| **Functional surgery (at least once) ¶** | 1.03 | [0.87-1.22] | 0.70 |  |  |  |  | |
| **Antipsychotic (at least once)** | 0.55 | [0.47-0.65] | < .001 *** |  | 0.75 | [0.63-0.89] | < .01 ** | |
| **Antidepressant (at least once)** | 1.11 | [1.03-1.20] | < .01 ** |  | 1.22 | [1.12-1.32] | < .001 *** | |
| **Anxiolytic (at least once)** | 0.93 | [0.86-1.00] | 0.06 |  |  |  |  | |
| **Z-drug (at least once)** | 0.92 | [0.84-1.01] | 0.09 |  |  |  |  | |
| **Antalgic (at least once)** | 0.94 | [0.87-1.01] | 0.10 |  |  |  |  | |
| **Thymoregulator (at least once)** | 0.49 | [0.33-0.73] | < .001 *** |  |  |  |  | |
| **Number of drugs #** |  |  |  |  |  |  |  | |
| No drug (year) | 0.48 | [0.38-0.59] | < .001 *** |  | 0.66 | [0.52-0.83] | < .01 ** | |
| Excessive polypharmacy (quarter) | 0.77 | [0.71-0.84] |  |  | 1.04 | [0.95-1.14] |  |  |
|  |  |  |  |  |  |  |  | |
| **Number of PIM #** |  |  |  |  |  |  |  | |
| Between one and five PIM | 1.23 | [1.12-1.36] | < .001 *** |  |  |  |  | |
| Between six and ten PIM | 1.07 | [0.91-1.26] |  |  |  |  |  | |
| Between 11 and 20 PIM | 0.91 | [0.78-1.05] |  |  |  |  |  | |
| More than 20 PIM | 0.81 | [0.67-0.97] |  |  |  |  |  | |
| **Medical transportation (at least once)** | 0.51 | [0.46-0.55] | < .001 *** |  | 0.73 | [0.66-0.81] | < .001 *** | |
| **Cane** | 0.75 | [0.62-0.92] | < .01 ** |  |  |  |  | |
| **Medical walker of wheelchair** | 0.44 | [0.36-0.54] | < .001 *** |  | 0.67 | [0.54-0.83] | < .001 *** | |
| **Anti-bedsore cushion or mattress** | 0.52 | [0.41-0.65] | < .001 *** |  |  |  |  | |
| **Patient lift or medical bed** | 0.47 | [0.39-0.58] | < .001 *** |  |  |  |  | |
| **Nutritional supplement** | 0.67 | [0.57-0.78] | < .001 *** |  | 0.80 | [0.68-0.95] | < .01 ** | |
|  |  |  |  |  |  |  |  | |
| **Interdepartmental variance** | 0.09 | [0.06-0.14] | < .001 *** |  | 0.10 | [0.06-0.15] | < .001 *** | |
| *PIM: Potentially Inappropriate Medication*  **ambulatory oncology, endocrinology, internal medicine, pulmonology consultations*  **†** *speech therapy, orthoptics, podiatry consultations*  **‡** *ambulatory dentist, gynecology, ophthalmology consultations*  **§** *flu vaccine, hearing test*  **¶** *cataract, total hip replacement, total knee replacement*  *# excluding antipsychotic, antidepressant, anxiolytic, z-drug, thymoregulator, antalgic* |  |  |  |  |  |  |  | |
