# Supplementary material for: Which factors preceding dementia identification impact future healthcare use trajectories: multilevel analyses in administrative data
Source: BMC Geriatr. 2024 Jan 23;24:89. doi: 10.1186/s12877-023-04643-1 (PMC10807194; doi:10.1186/s12877-023-04643-1)
Supplement: Supplementary file 6 — Additional file 6. Results of the multilevel multivariate analysis of factors associated with future favorable healthcare use trajectories of the 85 and older group (n=15,785). [file 12877_2023_4643_MOESM6_ESM.docx]

# Additional file 6: Results of the multilevel multivariate analysis of factors associated with a favorable future healthcare use trajectory of the 85 and older group (n=15,785)

|  | **85 and older group** | | | | | | |
| --- | --- | --- | --- | --- | --- | --- | --- |
|  | **(n=15,785)** | | | | | | |
|  | **Bivariate analysis** | | |  | **Multivariate analysis** | | |
|  | **OR** | **IC95%** | **p-value** |  | **aOR** | **IC95%** | **p-value** |
| **Sociodemographic caracteristics** |  |  |  |  |  |  |  |
| **Sex** |  |  |  |  |  |  |  |
| Female | 0.99 | [0.92-1.09] | 0.98 |  | 1.17 | [1.07-1.27] | < .01 ** |
| **Age (continuous)** | 0.87 | [0.86-0.88] | < .001 *** |  | 0.89 | [0.88-0.90] | < .001 *** |
| **Number of comorbidities (continuous)** | 0.93 | [0.91-0.96] | < .001 *** |  | 0.89 | [0.87-0.92] | < .001 *** |
| **Type of location of residence** |  |  |  |  |  |  |  |
| Rural location | 1.04 | [0.93-1.14] | 0.51 NS |  |  |  |  |
| **Deprivation index** |  |  |  |  |  |  |  |
| 2 | 0.98 | [0.87-1.10] | 0.03 * |  |  |  |  |
| 3 | 0.91 | [0.81-1.03] |  |  |  |  |  |
| 4 | 0.87 | [0.77-0.99] |  |  |  |  |  |
| 5 (the most deprived) | 0.84 | [0.74-0.95] |  |  |  |  |  |
| **Healthcare use before ADRD identification** |  |  |  |  |  |  |  |
| **Institutionalization** |  |  |  |  |  |  |  |
| Nursing home with internal pharmacy | 0.17 | [0.10-0.28] | < .001 *** |  | 0.25 | [0.14-0.41] | < .001 *** |
| Nursing home without internal pharmacy | 0.28 | [0.23-0.33] |  |  | 0.34 | [0.27-0.39] |  |
| **General Practicioner** |  |  |  |  |  |  |  |
| No or one GP consultation | 0.67 | [0.57-0.80] | < .001 *** |  |  |  |  |
| Between five and seven GP consultations | 1.23 | [1.09-1.38] |  |  |  |  |  |
| More than seven consultations | 1.18 | [1.06-1.31] |  |  |  |  |  |
| **Ambulatory nursing care** |  |  |  |  |  |  |  |
| Between once and four times | 1.45 | [1.32-1.59] | < .001 *** |  | 1.01 | [0.91-1.12] | < .01 ** |
| Five times and more | 1.27 | [1.17-1.38] |  |  | 0.83 | [0.75-0.92] |  |
| **Physiotherapy sessions** |  |  |  |  |  |  |  |
| Between one and ten sessions | 1.22 | [1.07-1.40] | 0.01 * |  |  |  |  |
| More than ten sessions | 1.00 | [0.92-1.09] |  |  |  |  |  |
| **Ambulatory cardiology consultation (at least once)** | 1.56 | [1.45-1.69] | < .001 *** |  | 1.28 | [1.18-1.39] | < .001 *** |
| **Ambulatory surgery consultation (at least once)** | 1.43 | [1.30-1.57] | < .001 *** |  | 1.13 | [1.01-1.26] | 0.03 * |
| **Ambulatory psychiatry consultation (at least once)** | 1.46 | [1.11-1.93] | < .01 ** |  |  |  |  |
| **Ambulatory neurology consultation (at least once)** | 2.00 | [1.32-3.02] | < .01 ** |  | 1.57 | [1.01-2.44] | 0.04 * |
| **Ambulatory dermatology/rheumatology/otorhinolaryngology consultation (at least once)** | 1.63 | [1.50-1.76] | < .001 *** |  | 1.23 | [1.13-1.34] | < .001 *** |
| **Ambulatory other medical specialty consultation (at least once) *** | 1.58 | [1.41-1.77] | < .001 *** |  | 1.26 | [1.11-1.42] | < .001 *** |
| **Ambulatory allied health professional consultation (at least once) †** | 1.44 | [1.25-1.66] | < .001 *** |  |  |  |  |
| **Outpatient consultation in hospital care (at least once)** | 1.15 | [1.07-1.23] | < .001 *** |  |  |  |  |
| **Prevention consultation (at least once) ‡** | 1.70 | [1.59-1.83] | < .001 *** |  | 1.29 | [1.19-1.39] | < .001 *** |
| **Preventive act (at least once) §** | 1.24 | [1.15-1.33] | < .001 *** |  | 1.11 | [1.03-1.20] | 0.01 * |
| **Ambulatory medical imaging (at least once)** | 1.52 | [1.42-1.63] | < .001 *** |  | 1.20 | [1.10-1.30] | < .001 *** |
| **Cumulated duration of planned hospitalization stay(s)** |  |  |  |  |  |  |  |
| Between one and five days | 1.26 | [1.07-1.48] | 0.01 * |  |  |  |  |
| More than five days | 0.96 | [0.87-1.06] |  |  |  |  |  |
| **Planned short hospitalization (same entry and exit date)** |  |  |  |  |  |  |  |
| Once | 1.67 | [1.44-1.94] | < .001 *** |  | 1.33 | [1.14-1.56] | < .01 ** |
| At least twice | 1.43 | [1.15-1.79] |  |  | 1.04 | [0.82-1.31] |  |
| **Emergency room visit without hospitalization (at least once)** | 0.91 | [0.82-1.01] | 0.06 |  |  |  |  |
| **Unplanned hospitalization (via the emergency room) (at least once)** | 0.84 | [0.77-0.92] | < .001 *** |  |  |  |  |
| **Potentially avoidable hospitalization (at least once)** | 0.83 | [0.70-0.98] | 0.031 * |  |  |  |  |
| **Hospitalization with neuropsychiatric disorder (at least once)** | 0.78 | [0.58-1.05] | 0.10 |  |  |  |  |
| **Functional surgery (at least once) ¶** | 1.53 | [1.27-1.85] | < .001 *** |  |  |  |  |
| **Antipsychotic (at least once)** | 0.73 | [0.63-0.85] | < .001 *** |  |  |  |  |
| **Antidepressant (at least once)** | 1.09 | [1.02-1.18] | 0.02 * |  |  |  |  |
| **Anxiolytic (at least once)** | 1.12 | [1.04-1.20] | < .01 ** |  |  |  |  |
| **Z-drug (at least once)** | 1.17 | [1.07-1.27] | < .01 ** |  | 1.16 | [1.05-1.27] | < .01 ** |
| **Antalgic (at least once)** | 1.30 | [1.21-1.40] | < .001 *** |  | 1.19 | [1.10-1.29] | < .001 *** |
| **Thymoregulator (at least once)** | 1.18 | [0.71-1.97] | 0.52 |  |  |  |  |
| **Number of drugs #** |  |  |  |  |  |  |  |
| No drug (year) | 0.48 | [0.39-0.58] | < 0.001 *** |  | 0.75 | [0.61-0.93] | < .001 *** |
| Excessive polypharmacy (quarter) | 1.30 | [1.21-1.40] |  |  | 1.19 | [1.09-1.30] |  |
|  |  |  |  |  |  |  |  |
|  |  |  |  |  |  |  |  |
| **Number of PIM #** |  |  |  |  |  |  |  |
| Between one and five PIM | 1.27 | [1.16-1.40] | < .001 *** |  |  |  |  |
| Between six and ten PIM | 1.44 | [1.24-1.68] |  |  |  |  |  |
| Between 11 and 20 PIM | 1.21 | [1.06-1.38] |  |  |  |  |  |
| More than 20 PIM | 1.58 | [1.34-1.87] |  |  |  |  |  |
| **Medical transportation (at least once)** | 0.82 | [0.76-0.89] | < .001 *** |  | 0.846 | [0.775-0.923] | < .001 *** |
| **Cane** | 1.34 | [1.12-1.60] | < .01 ** |  |  |  |  |
| **Medical walker of wheelchair** | 0.81 | [0.71-0.92] | < .01 ** |  |  |  |  |
| **Anti-bedsore cushion or mattress** | 0.76 | [0.65-0.91] | < .01 ** |  |  |  |  |
| **Patient lift or medical bed** | 0.68 | [0.59-0.79] | < .001 *** |  | 0.780 | [0.671-0.905] | < .01 ** |
| **Nutritional supplement** | 0.76 | [0.68-0.8475 | < .001 *** |  | 0.761 | [0.677-0.856] | < .001 *** |
|  |  |  |  |  |  |  |  |
| **Interdepartmental variance** | 0.04 | [0.02-0.06] | < .001 *** |  | 0.032 | [0.017-0.058] | < .001 *** |
| *PIM: Potentially Inappropriate Medication*  **ambulatory oncology, endocrinology, internal medicine, pulmonology consultations*  **†** *speech therapy, orthoptics, podiatry consultations*  **‡** *ambulatory dentist, gynecology, ophthalmology consultations*  **§** *flu vaccine, hearing test*  **¶** *cataract, total hip replacement, total knee replacement*  *# excluding antipsychotic, antidepressant, anxiolytic, z-drug, thymoregulator, antalgic* |  |  |  |  |  |  |  |
